# Impact of Cannabis and Cannabis Legalization on US Atrial Septal Defect Rates

**DOI:** 10.3390/jox16020043

**Published:** 2026-03-01

**Authors:** Albert Stuart Reece, Gary Kenneth Hulse

**Affiliations:** 1 Division of Psychiatry, University of Western Australia, 35 Stirling Hwy., Crawley, WA 6009, Australia; gary.hulse@uwa.edu.au; 2 School of Medical and Health Sciences, Edith Cowan University, 27 Joondalup Dr., Joondalup, WA 6027, Australia

**Keywords:** cannabis, cannabinoid, congenital anomalies, cardiogenesis, genotoxicity, epigenotoxicity, transgenerational inheritance, teratogenesis, multigenerational

## Abstract

Atrial septal defect (ASD) affects 1:11.3 children in some US states; however, the antecedents of these trends are yet to be identified. A total of 1882 ASD rates (ASDRs) for 2003–2020 were sourced from the National Birth Defects Prevention Network reports. A total of 406,893 ASDs are reported. Substance (cigarettes, binge alcohol, cannabis, cannabinoids, analgesics, cocaine) exposure data were taken from the National Survey of Drug Use and Health. Income and ethnicity data were derived from the US Census. Adjustment was performed by mixed effects, survey and generalized additive regression. Causal analysis was by inverse probability weighting and E-values. Data were analyzed in RStudio. The highest ASDR of 884/10,000 live births was amongst Non-Hispanic Asians and Pacific Islanders in Nevada in 2016–2020. The 2005–2018 median ASDR rose >12-fold in Nevada and New Mexico, >6-fold in New York, and 4.2-fold nationally 1989–2020; it doubled in NY from 2012–2016 to 2016–2020. The average state ASDR rose supra-exponentially (*p* = 0.0075) and was associated with higher cannabis use states (*p* = Zero, Cohen’s D = 1.24), apparently driven by cannabis legalization (*p* = Zero). Estimated exposures to Δ9THC, cannabidiol and cannabigerol were implicated (from *p* = 2.67 × 10^–68^). Cannabis-legal states were compared with others (mean ASDR (C.I.) 178.15 (131.68, 224.62) vs. 74.28 (70.60, 77.96), *p* = Zero; O.R. 1.82 (1.81, 1.84), E-values 3.04 (lower C.I. 3.02), Cohen’s D 1.29 (0.96, 1.62)). Overall, 29/39 (74.4%) E-value estimates were >4; 39/39 (100%) were >1.25. Cannabis, cannabinoids and cannabis legalization are strong candidates for driving the US ASDR supra-exponentially. Estimates of many cannabinoids, including cannabidiol, Δ9THC, and cannabigerol, are implicated. The results are consistent with other large epidemiological studies. The importance of the results is magnified by the increasing legalization and penetration of cannabinoids into the US population. Since therapeutic abortion is not practiced for ASD, it may be used as a bellwether index of heritable transgenerational cannabinoid genotoxicity and epigenotoxicity associated with cannabinoid exposure.

## 1. Introduction

Whilst much recent attention has focused on the severe negative impacts of cannabinoids on brain development [1,2,3,4,5,6], the cardiovascular impacts of cannabis are also well described, albeit less widely recognized [2,7,8,9,10,11].

Recent reports of atrial septal defect (ASD) secundum rates (ASDRs) show peak prevalence at 884/10,000 live births amongst Non-Hispanic Asians and Pacific Islanders (NHAsPI) 2016–2020 in Nevada [12]. Surprisingly, this daunting modern trend has not been explored by contemporary researchers, perhaps in part because the official ASDR figure quoted by CDC is 10.3/10,000 based upon data from Atlanta, Georgia, 1998–2005, which is one of the lowest incidence states [13,14].

ASD is an important disorder for several reasons. Atrial septal defect is the commonest of congenital cardiac anomalies, which are, in turn, the commonest of congenital anomalies. Early therapeutic termination of pregnancy for anomaly (ETOPFA) is not practiced for ASD as it is for more major congenital anomalies, making its prevalence data more reliable than for more serious defects, where reports of accurate ETOPFA rates are sparse. Several studies have now demonstrated a causal link between cannabis and ASD (C-ASD) [10,15,16,17,18,19,20] so that ASD is a useful biomarker not only of cannabis teratogenicity but also of cannabinoid genotoxicity more broadly. Several cannabinoids are known to have an exponential genotoxic dose–response curve [1,2,3,4]. Further, since ASD is a congenital anomaly, it represents intergenerational transmission of cannabinoid genotoxicity potentially via epigenetic inheritance [10,20,21,22,23,24].

ASD was first reported to be linked with cannabis exposure by Hawaiian epidemiologists who noted a rate ratio of 6.12 (95%C.I. 1.98, 14.35) [16]. This result is associated with an E-value estimate of 11.72, which is even stronger than the effect of tobacco on lung cancer (E-value of 9), with a 95% lower bound of 3.37, indicating both that causality is highly likely and that spurious explanation of the link by extraneous confounding is unlikely [25,26]. More recent investigations of USA data showed a close relationship across space and time between ASDRs and cannabis exposure and that the relationship fulfilled mechanistic and mathematical criteria for causality [5,15,20]. Previous laboratory and population-based studies demonstrated exponential dose–response relationships between cannabis exposure and ASDRs and many congenital anomalies [20]. Collectively, the association of cannabis with ASDRs has been confirmed by large population-based studies from Hawaii, Canada, Australia, Colorado, USA and Europe [10,15,16,17,18,19,20].

The pre-specified purposes tested by this study were: (1) to describe and dissect recent trends in atrial septal defects; (2) to explain their associations and causes; (3) to examine the impact of cannabinoids on ASDRs; and (4) to examine the effect of cannabis legalization on ASDRs.

## 2. Methods

### 2.1. Data

#### 2.1.1. Congenital Anomalies

The complete dataset relating to atrial septal defect (secundum type) was downloaded from the Annual reports of the National Births Defects Prevention Network (NBDPN) [6], which is affiliated with the Centers for Disease Control, Atlanta, Georgia, USA. These data related to the rates by all ethnicities for all the years listed. Ethnicities studied were as listed in the NBDPN dataset, which were Non-Hispanic White (NHWhite), Non-Hispanic African American (NHBlack), Hispanic, Non-Hispanic Asian–Pacific Islanders (NHAsPI), Non-Hispanic American Indian/Alaskan Native (NHAIAN) and Total (Overall). The first available data was for the 1989–1990 biennium. Most of the data periods were over five years. The most recent was that for 2016–2020. In such cases, the central year (here, 2018) was taken as the “Indicative Year” and used for data analysis and display purposes in graphs. For periods such as biennia 1989–1990, the second year was used as the Indicative Year. Data for atrial septal defect rates appeared in 12 Annual Reports across this period. Ten of these datasets overlapped with the drug datasets (2005–2018), which were accordingly used in regression analyses. All available data were used. Missing data were casewise deleted.

#### 2.1.2. Drugs

Drug use data by state for the period 2005–2018 were taken from the Restricted Use Dataset (RUD) of the National Survey of Drug Use and Health (NSDUH), conducted annually by the Substance Abuse and Mental Health Services Administration (SAMHSA) [7]. Indices of interest included the last month use of cigarettes (cigmon), the last month use of alcohol (alcmon), binge drinking in the last month (bngalc or bngdrkmon), alcohol use disorder in the past year (audyr, depndalc), the last month use of cannabis (mrjmon), the last year use of opioid analgesics (anlyr, pnrnmyr) and the last year use of cocaine (cocyr). The quanta for the covariate pairs of bngalc and bingdrkmon and for anlyr and pnrnmyr were not directly comparable due to changes in the survey questions applied in different iterations of the NSDUH. Small arithmetical corrections were applied to the second data series to induce direct comparability such that the last value of the mean of the two datasets nationally was comparable to the first mean value of the following dataset. NSDUH data collection was impacted by the COVID-19 pandemic and was reflected in part in these nomenclature differences.

#### 2.1.3. Others

Data on population ethnicity and median income were downloaded from the US Census Bureau using the censusapi package in R Studio [8]. Data on the cannabinoid concentration of cannabis samples were derived from published reports of analysis of cannabis seizures by the Drug Enforcement Agency (DEA) [9,10,11,12,13]. Data relating to the percentage rate of high potency seizures (exceeding 12% Δ9THC (tetrahydrocannabinol) content) were also taken from recently published reports [10]. Data relating to the legal status of cannabis were taken from online resources [14,15,16]. The number of births in each state was taken from the CDC WONDER database [17].

#### 2.1.4. Derived Variables

Percentage rates of ethnic drug use taken from the NSDUH were standardized against the national population overall for that year as a simple ratio. These national ratios were then multiplied by the state ratios of percent ethnicity to define an average index of ethnic drug exposure for that state. States were divided into high- and low-cannabis-exposure states by designating states in the top (numerical) tertile of cannabis use in the most recent drug sampling year (2018) as high-cannabis-exposure states. This incorporated the states of Alaska, Colorado, Maine, Massachusetts, Michigan, Nevada, New Hampshire, New Mexico, Oregon, Rhode Island and Vermont. The Δ9THC content was multiplied by the monthly cannabis use rate to derive a compound variable (d9THC_mrjmon). This parameter was then multiplied by the frequency of high-THC-potency seizures (>12%) to derive a further compound variable (d9THC_HiPot_mrjmon).

### 2.2. Data Analysis

Data analysis was conducted in R version 4.4.3 from the Central R Archive Network (CRAN) and performed in R Studio (2024.12.1 Build 563). Data were manipulated using dplyr from tidyverse (version 2.0.0) [18], and graphs were drawn in ggplot2, also from tidyverse, aided by ggpubr (version 0.6.0) [19], ggbreaks (version 0.1.4) [20], ggrepel (version 0.9.6) [21], patchwork (version 1.3.0) [22], grid (version 4.5.1 from R Base) and gridExtra (version2.3) [23]. Table 1 was compiled using the tableone package (version 0.13.2) [24]. Covariates such as ASD rate, last month cannabis use (mrjmon), annual analgesic abuse, annual cocaine abuse and median income were log-transformed in the interests of normality assumptions.

Regression models were fitted as appropriate using lme4 (version 1.1-37) [25] and lmerTest (version 3.1-3) [26], survey (version 4.4-2) [27,28], and the general additive model (gam) function from mgcv (version 1.9-3) [29,30,31], incorporating the negative binomial distribution function (negbin) from MASS (version 7.3-65) [32]. In models including ethnicity, the comparator population was NHAsPI unless otherwise noted. In mixed effects models, state was assigned as the random effect. Since data were sampled from similar populations over time, it was also appropriate to use survey glm (svyglm, generalized linear models) regression. In survey glm models, the design included state as a stratum. In view of the fact that ASD is notionally a rare event with rates related to underlying count data, the negative binomial distribution was chosen for the generalized additive models (mgcv::gam) with theta set to unity. The offset chosen was the number of births in each state. Parametric terms were smoothed. A smoothed term including time and state was also included in all gam models. Interaction terms in gam models were tensor product (te) or tensor product interactions (ti), which include lower-order effects, as indicated. Models became progressively more complex within each group, including the inclusion of more covariates, more cannabinoids and more interactive terms.

Models were compared by reviewing their Akaike Information Criterion (AIC) and directly via anova tests, as appropriate. The Bayesian Information Criterion (BIC) and Log Likelihood ratio (logLik) were also calculated. For each regression type, final models were obtained from initial full models by the classical method of the serial removal of the least significant term.

Multicollinearity among covariates was assessed using variance inflation factors (VIFs) using the R packages car (version 3.1-3) and performance (version 0.14.0) [33,34]. For models including multilevel categorical variables, generalized variance inflation factors (GVIFs) were calculated and adjusted using the standard transformation GVIF^(1/(2×df))^ to allow comparison with conventional VIF thresholds. Race was included as a social covariate reflecting structural and contextual factors rather than biological differences. Collinearity diagnostics were evaluated for the fixed-effects portion of mixed models, consistent with standard practice for mixed-effects regression.

Effect size was calculated using Cohen’s D derived from the R packages effectsize and emmeans (1.11.1) [35,36]. All t-tests were two-sided. *p* < 0.05 was considered significant.

### 2.3. Causal Inference

All mixed-effects, survey glm and gam models were inverse probability weighted using the ipwt function from the ipw package (version 1.2.1) [37]. E-values were calculated using the EValue package (version 4.1.3) [38,39,40] from regression models and bivariate comparisons to explore the magnitude of potentially causal relationships, taking 1.25 as the cut-off for causal association of the E-value estimate [38] and values of above 9 to be very elevated [39].

### 2.4. Ethics Approval

Ethical approval for this study was provided by the Human Research Ethics Committee of the University of Western Australia, number RA/4/20/4724, on 24 September 2021.

### 2.5. Generative Artificial Intelligence

Generative artificial intelligence was not used in the generation of this paper.

### 2.6. Data Availability

The data and computational code are openly available via Mendeley at doi: https://doi.org/10.17632/ny2j3msk86.2.

## 3. Results

### 3.1. ASD Rates

A total of 2251 ASDRs were obtained for the 1989–2020 period, of which 1882 fell in the period 2005–2018 when both congenital anomaly and drug use data were available. Table 1 shows the social and demographic data for the 2005–2018 dataset stratified into high- and low-cannabis-using states as described in the Methods. A total of 406,893 ASD cases were reported amongst 65,618,252 births (counting Hispanics) across 46 states. Many significant differences are shown. Interestingly, the legal status across the two sets of states is highly significantly different (ChiSqu.Trend = 1503.6, *p* = 7.33 × 10^−321^).

**Figure 1:**
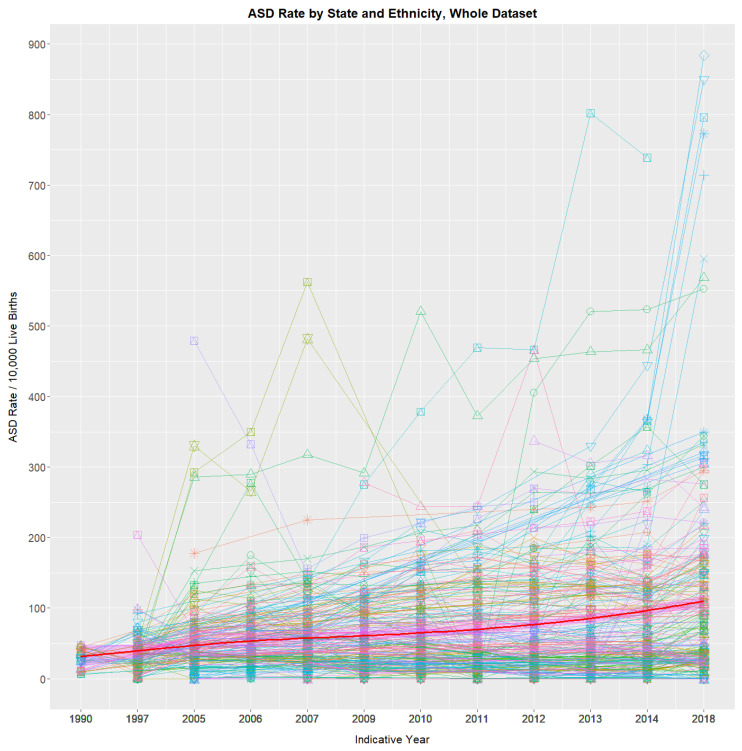
ASD rates by state and ethnicity. A total of 276 state–ethnicity rates are shown. Detailed explanations of the lines and colours are shown in Figures S2–25.

Figure 1 shows the trend over time of the 276 state–race combinations, together with the average. A general upward trend is noted. Table S1 shows the 12 time periods, including their Indicative Years for which NBDPN ASDR data are available from 1989 to 2020, together with the mean rates across all state and ethnicity combinations for each year. A 4.23-fold rise from 27.4/10,000 live births to 116.0 across this period is observed.

A straight line appearing on a log plot depicts an exponential rate of growth. Concerningly, when state rates are charted in this way, rates in states such as Nevada and New Mexico curve concave upwards on a log plot, which is indicative of supra-exponential increases (Figure S1).

ASDRs by ethnicity for each state are shown paneled in Figures S2 and S3. Figures S4–S16 illustrate detailed presentations of high ASD states, including Nevada, New York, Department of Defense, Kentucky, Michigan, Colorado, Tennessee, Alaska, Mississippi, Missouri, New Mexico and Oregon. The 2005–2018 ASDR has risen a median of 12.21-fold in Nevada (Figure S4; *p* = 7.95 × 10^−6^; overall ethnicity 67.82 to 772.8), 6.05-fold in New York (Figure S5; *p* = 4.9 × 10^−4^), 2.32-fold in the Department of Defense (Figure S7; *p* = 5.21 × 10^−6^), 3.8-fold in Colorado (Figure S10; *p* = 2.25 × 10^−4^), 12.48-fold in New Mexico (Figure S15, from 25.35 to 316.5, *p* = 5.39 × 10^−6^) and 1.99-fold in New York for just 2014–2018 (Figure S6; *p* = 1.9 × 10^−4^). The highest rate reported was 884 amongst Non-Hispanic Asians/Pacific Islanders in Nevada in 2016–2020.

Figures S17–S25 show ASDRs in various low-ASD states, including Florida, Utah, Texas, Georgia, Maryland, Minnesota, Iowa, South Carolina and New Jersey, which had rates in the most recent reporting period of 114.9, 32.2, 89.9, 25.8, 12.1, 27.8, 23.6, 9.2, and 39.0.

### 3.2. Bifurcation of ASD Rates

**Figure 2:**
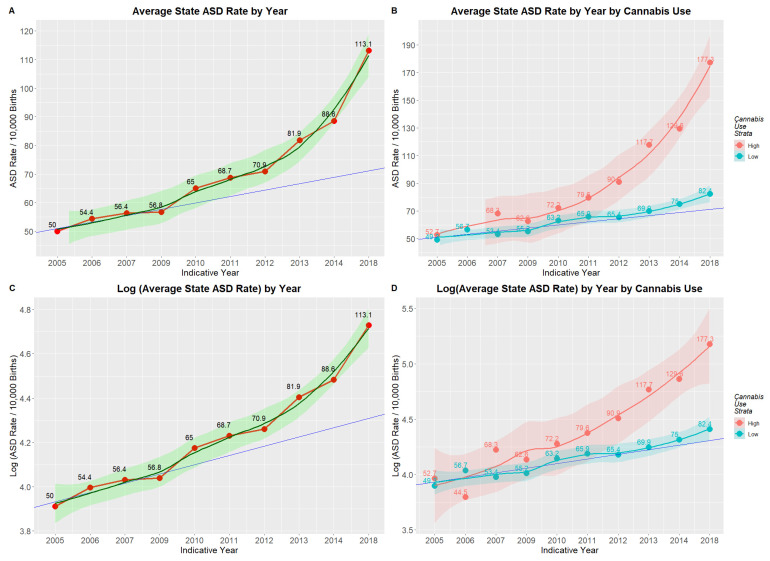
(**A**) Mean state ASD rates by year; (**B**) log (mean state ASD rates) by year; (**C**) mean state ASD rates by year by cannabis use rates in 2018; (**D**) log (mean state ASD rates) by year by cannabis use rates in 2018.

Figure 2A,C show the mean annual state ASDR on linear and logarithmic plots, respectively, together with regression lines fitted to their first four years. A clear departure is shown in each case from the linear trend, which, in the second case, is therefore supra-exponential. When models linear and quadratic in time are fitted to log ASDRs, the quadratic model demonstrates superior fit, confirming the visual appearance (AIC linear −21.14, AIC quadratic −30.04; Anova F = 13.82, *p* = 0.0075).

Figure 2B,D show that this departure from linearity is wholly attributable to rates in the high-cannabis-use states. A gam model shows that the divergence between high- and low-cannabis-using states has not arisen by chance (edf = 0.984, F = 62.77, *p* = Zero; model: AdjRSqu. = 0.323, GCV = 1.1531, n = 1882). In a mixed-effects model, Cohen’s D for this effect is 1.24 (C.I. 0.21, 2.27).

### 3.3. Substance Use

Figures S26–S29 illustrate US substance use. Most substance use has declined or is stationary, the sole exception being cannabis, which has risen 1.91-fold from 5.81% to 11.08%. National reports list the rate of high THC cannabis seizures and their THC concentration. When these are multiplied by monthly cannabis use, an index can be derived, which has risen 6.6-fold (1567.8 to 10,320.5; Table S2). Exposure to many cannabinoids has risen (Figure S30).

### 3.4. ASDR over Time vs. ASDR by Cannabis Exposure

Figure S31A–D show that the slope of the ASD by time line and the slope of the ASD by cannabis exposure line are very closely related (β-est. = 0.435, t = 15.24, *p* = 5.06 × 10^−18^; model Adj.R.Squ. = 0.853). They also show that in 2012–2018, more states at the high end of the regression zone moved to medical or legal cannabis.

### 3.5. Multivariate Modeling

#### 3.5.1. Mixed-Effects Models

In multivariate mixed-effects modeling of cannabis use, Δ9THC and cannabigerol concentration were shown to be closely related to ASDRs (first five models; Table S3). When all the substances and incomes were considered, cannabis was the most significant term. Ethnicity was also significant, but its relative significance declined when ethnic drug exposure was considered. Cannabis interacted significantly with several ethnicities. In the final interactive model, cannabis was the most significant term. Increasingly complex mixed-effects regression models are shown in Table 2. Cannabis use is significant in all models. The final interactive model uses ethnic substance exposure and is not weighted to allow effect size calculation. Partial effect sizes are presented in Table S4 and Figure S32. Only terms involving ethnic cannabis use are found to significantly contribute to model power. Table S5 summarizes these data by covariate grouping, and cannabis is shown to have much higher total partial variance than cigarettes and ethnicity (9.0% vs. 1.7% and 1.2%, respectively, with seven, three and one terms each). 

#### 3.5.2. Survey Regression Models

Survey regression was conducted to account for the temporal overlap in the data. Cannabis and cannabinoids Δ9THC, cannabidiol and cannabigerol were significant in increasingly complex models (Table S6). Generalized additive models were performed to account for the curvilinear nature of the data. Again, cannabis and cannabinoids were found to be highly significant in all models (Table S7). Nine *p*-values listed as Zero in this table signify *p* < 10^−321^.

### 3.6. Legal Status

**Figure 3:**
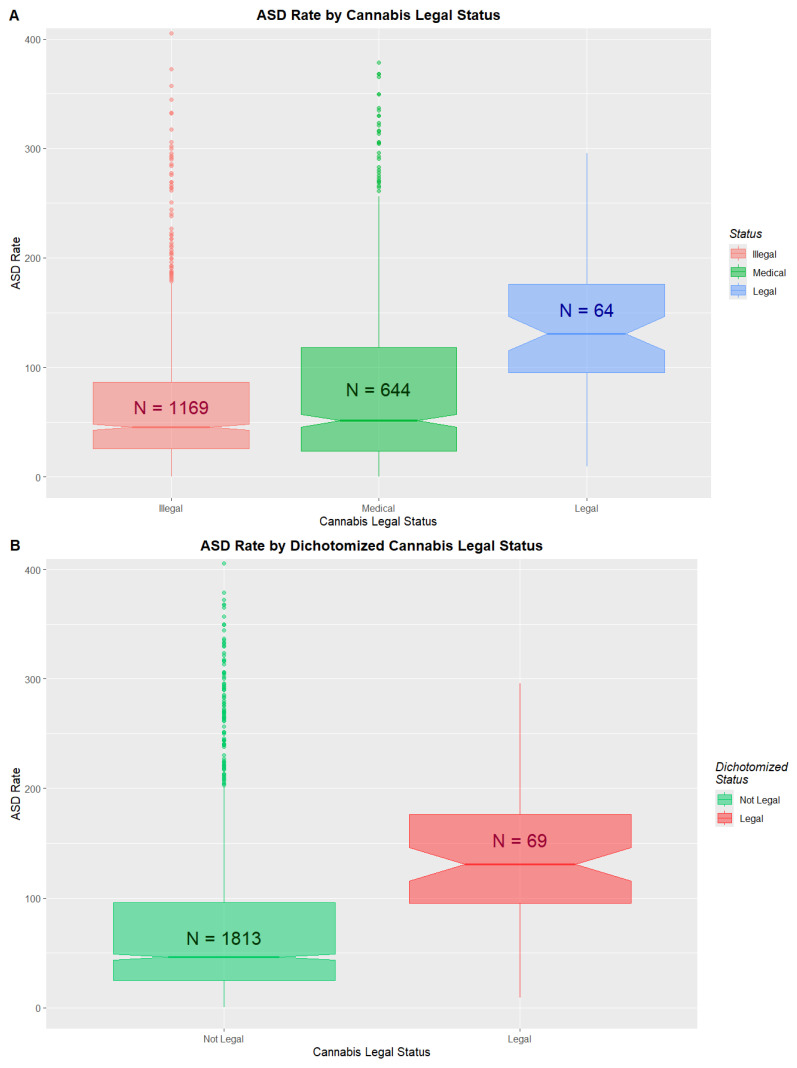
(**A**) ASD rates by cannabis legal status; (**B**) ASD rates by cannabis legal status dichotomized as states where cannabis was legal vs. others.

The changing legal status of cannabis across the USA is illustrated in Figure S33. ASDRs by legal status are shown in Figure 3 and Table S8. Medical and decriminalized have been combined here due to the small numbers in the decriminalized group. A clear trend to increased ASDRs with liberal cannabis paradigms is shown (ChiSqu.Trend = 2724, *p* = 0.0023; Figure 3A). When the data are dichotomized into ASDRs in legal states v others (mean (C.I.) 178.15 (131.68, 224.62) vs. 74.28 (70.60, 77.96)), significant differences are found (ChiSqu. = 22,194, *p* = Zero; O.R. 1.82 (1.81, 1.84)), Attributable Fraction in the Exposed 44.92% (44.48%, 45.35%), E-values 3.04 (lower C.I. 3.02) and number needed to harm 1 in 48 (48, 49) (Figure 3B).

### 3.7. Effect Sizes

Cohen’s D for the bivariate pairwise comparisons by legal status are shown in Table S9. Many are very high (legal–illegal 1.29 (0.96, 1.62), legal–decriminalized 1.10 (0.54, 1.66); interpretive note: Cohen’s D values > 0.8 are said to be large; those > 1.2 are said to be very large).

### 3.8. Within- and Between-States Mixed-Effects Modeling

A hybrid mixed-effects model was constructed, which includes terms for within-state and between-state cannabis variation separately using state–ethnicity as the random effect. Table S10 presents bivariate, additive and interactive hybrid models along with cannabis legal status. Progressively increasing model complexity and the contribution of cannabis variance rapidly negates the effects of cannabis legal status and increases the marginal variance attributable to measured covariates from 2.8% to 5.6% to 6.8%. Within-state variance was about twice as powerful a predictor as between-state variance. The final model explained 71.9% of the variance, mostly due to state–ethnicity contextual structure.

Cohens D’s from the pairwise comparisons in these hybrid models are presented (Table S11).

Cohen’s D similarly collapses with the inclusion of increasing cannabis variance: for the legal–illegal comparison, this falls from 1.29 to 0.43 to 0.06 (N.S).

### 3.9. Sensitivity Analysis

Many E-values can be calculated from these results (Table S12). E-value estimates and lower bounds were (median (IQR) 5.00 (4.00, 43.00)) and (4.00 (3.00, 10.00). Overall, 14/39 (35.9%), 29/39 (74.4%) and 39/39 (100%) of the E-value estimates are in the high (>9), moderate (>4) and causal (>1.25) ranges, respectively (Table S12). For the 95% E-value lower bounds, 11/39 (28.2%), 20/39 (51.3%) and 39/39 (100%) fall in these same ranges.

### 3.10. Variance Inflation Factors

Variance inflation factors may be assessed to assess potential covariate collinearity. Across all mixed-effects model specifications, there was no evidence of problematic multicollinearity (Table S13 refers to the mixed-effects models shown in Table 2). Collinearity diagnostics based on variance inflation factors (VIFs) and adjusted generalized VIFs (GVIFs) indicated consistently low to moderate correlations among fixed-effect predictors. In the primary mixed models (Models 1–3 in Table 2; VIFs shown in Table S13), all adjusted VIF values were below 2, with tolerance values well above commonly accepted thresholds, indicating low collinearity. In the interaction model (Model 4 in Table 2), which included higher-order terms and multilevel categorical variables, predictor-level adjusted GVIFs (GVIF^(1/(2×df))^) remained below 3 for all predictors, including race and substance use variables. Elevated raw GVIF values observed for some terms reflected model complexity and interaction structure rather than substantive multicollinearity. Although race participated in interaction terms, adjusted GVIF values for race were low across all models, indicating that race was not excessively collinear with socioeconomic or substance use predictors. Overall, collinearity diagnostics supported stable estimation of fixed effects across all model specifications.

## 4. Discussion

### 4.1. Main Results

The main findings were that the mean state ASDR has increased >four-fold from 27.4 to 116.0 in the 1989–2020 period, often increasing from rare to common in many states. The peak rate was NHAsPI in Nevada at 884.0/10,000. The state averages show a supra-exponential distribution, which is wholly attributable to high-cannabis-use states, which are increasingly dominated by liberal cannabinoid legal paradigms. The ASDRs in Nevada and New Mexico have each increased >12-fold, and in New York, the ASDR increased >6-fold. Substance exposure across the USA was generally stationary or declining, except for cannabis, which rose 1.9-fold. One ΔTHC exposure index rose 6.6-fold in 2009–2018. In a fully adjusted mixed model, cannabis was a much more powerful predictor than either tobacco or ethnicity. Cannabis was significant when considered as both a within- and between-states covariate. The results were cross-validated by inverse probability weighted mixed-effects, survey and generalized additive regression. The ASDR in states where cannabis was legal was much higher than elsewhere (O.R. 1.82 (1.81, 1.84); Cohen’s D 1.29 (0.96, 1.62)). C-ASD E-values were generally moderately elevated, which points towards strong associations and makes uncontrolled confounding unlikely.

### 4.2. Interpretation

These results strongly support the strong association of cannabis with ASDRs both mechanistically and epidemiologically, which is consistent with a number of large studies elsewhere [5,41,42,43,44,45,46].

Genotoxicity has been well established as a foundational issue in drug safety testing since the thalidomide “tragedy” of 1957. Genotoxicity is manifested clinically as congenital anomalies, cancerogenesis, mental retardation and aging.

### 4.3. Literature Review

The databases PubMed, Medline, Scopus, Embase, Web of Science, Toxline, Mendeley, Current Contents, Biomed Central, Elsevier, and Springer were searched for the terms “cannabis”, “marijuana” and “atrial septal defect” (ASD) across all languages. Seven studies were identified, providing moderate- to high-quality data, often fulfilling causal criteria mechanistically, algorithmically and analytically.

### 4.4. Cellular and Molecular Mechanisms

#### 4.4.1. Morphological Cardiogenesis

Morphogenesis of central cardiovasculature is a complex, delicately orchestrated process involving cells from the primary, secondary and lateral heart fields and proepicardium, nuchal crest and parts of the pharyngeal arches [47]. Atrial septal formation involves the formation of a septum primum, its subsequent breakdown, the growth of the atrial septum secundum from the dorsal atrial roof, flap valve closure of the foramen ovale with life’s first breath and later anatomical fusion of the septa secundum and primum remnants in the first year of life. This complex cellular choreography is controlled by morphogen gradients, which signal to cardiogenic gene cassettes under epigenomic control. Clearly, anything that perturbs or disrupts the morphogen gradients, the genome or the epigenome can potentially have a large impact on both cardiogenesis and central vasculogenesis.

#### 4.4.2. Cardiogenic Morphogens

Morphogens are signaling molecules that play a crucial role in the embryonic development of the heart in mammals. They form concentration gradients within developing tissues, guiding the differentiation and spatial organization of cells to ensure proper cardiac formation. Many primary morphogens are involved in heart development, including sonic hedgehog (SHH), bone morphogenetic proteins (BMPs), Lefty proteins, components of the Wnt signaling pathway, COUP-TF II (Chicken Ovalbumin Upstream Promoter Transcription Factor II), VEGF (Vascular Endothelial Growth Factor) and VEGFR, Eph, angiopoietin and notch [47]. Cannabis impairs sonic hedgehog function both directly [3,48,49,50] and epigenomically [51]. Cannabis impairs Wnt signaling directly [52,53,54,55,56,57,58] and epigenomically [51]. Cannabinoids impair BMP signaling directly [59,60,61,62] and epigenomically [51]. Cannabis inhibits COUP-TF II directly [63]. Cannabinoids interact with angiopoietin pathways directly [64,65,66,67,68]. Cannabinoids interact with notch both directly [69,70,71] and epigenomically [51]. A total of 427 epigenomic hits against Eph genes were noted in the North Carolina epigenome-wide association study (EWAS) [51].

Therefore, the conclusion that cannabinoids broadly disrupt cardiogenic morphogenic gradients in multiple dimensions becomes inescapable.

It is of interest to consider the retinoid pathway by way of example for one of these pathways specifically.

Retinoids, derivatives of vitamin A, play essential roles in cardiac development, influencing gene expression via retinoic acid (RA) signaling. During early embryogenesis, RA regulates the patterning of the anterior–posterior axis of the heart tube and contributes to chamber specification, myocardial proliferation, and outflow tract development [72]. RA signaling is mediated through nuclear RA receptors (RARs) and retinoid X receptors (RXRs), which modulate transcription of key developmental genes such as *Tbx5*, *Nkx2.5*, and *Hox* family members [73]. Disruption of RA signaling, whether through deficiency or excess, leads to congenital heart defects (CHDs), highlighting its dosage-sensitive role in cardiogenesis [74].

Specifically, retinoids are crucial in atrial septal development [75,76]. The atrial septum arises from multiple embryonic structures, including the septum primum and septum secundum, whose formation is regulated by RA signaling. RA is required for the correct expression of *Tbx5*, a transcription factor critical for septation of the atria [77]. Mouse models with impaired RA synthesis (e.g., *Raldh2*-null mice) or disrupted RAR function exhibit atrial septal defects (ASDs), indicating a direct role in atrial partitioning [78]. Furthermore, RA influences endocardial cushion formation and remodeling processes that are essential for complete septation [79].

Taken together, retinoids are indispensable for proper heart development, particularly in atrial septation. Both deficiency and excess of RA disrupt normal morphogenesis, emphasizing the need for tightly regulated RA signaling during cardiogenesis.

Whilst cannabinoid products are an emerging industry, cannabis has long been known to be directly toxic to genetic material, including the induction of breaks in DNA and chromosomes, nuclear blebs, nucleocytolasmic bridges and multipolar cell division. As these are also indices of genomic aging, this evidence also speaks to nuclear aging. Increasing attention has recently focused on widespread epigenomic disruption induced by cannabis intoxication and withdrawal relating to all body systems and fundamental cellular processes, including DNA maintenance, formation and control of the mitotic spindle, reading, writing and erasing of DNA methylation, histone synthesis and metabolism, cellular and organismal growth and mitochondrial metabolism. Since aging has now been shown to be caused by genomic and epigenomic disruption, this evidence also implies cellular and organismal aging [80,81,82,83]. Indeed, cannabis has been shown to drive aging at both the epigenomic and clinical levels and in eighteen other domains [84,85].

Concerningly, several studies implicate many cannabinoids based upon the genotoxicity of the olivetol ring on the C-ring common to all cannabinoid structures [86].

#### 4.4.3. Cardiogenic Epigenomics

This subject has already been reviewed in detail elsewhere [5,44,84,85]. The following material is included to provide an introductory appreciation of the scope and depth of the cardiogenic epigenotoxic damage inflicted by cannabis and cannabinoids. The interested reader is referred to the published literature and the primary cited sources [51].

In chronicling over 20,000 cannabis-induced perturbations of human and rodent sperm DNA methylation, one recent epigenome-wide screen (EWAS) found that amongst the most impacted pathways and processes perturbed by cannabis dependence, cardiogenesis was ranked fifth, whilst neurodevelopmental disorders and cerebral disorders were ranked ninth and tenth [51]. Similarly, disruption of vasculogenesis was also featured in the top ten processes most disrupted by cannabis withdrawal [51]. Organismal growth and agenesis pathways were ranked sixth and seventh in cannabis dependence, and organismal death was ranked fifth in cannabis withdrawal [51]. Moreover, some of the key genes regulating heart development, including GATA and TBX, were also shown to be epigenomically disrupted [51].

As mentioned, cannabinoids interfere with the major readers, writers and erasers of the epigenomic machinery epigenomically [5,51,84,85,87]. In the case of TET1, the major eraser of DNA methylation, cannabidiol and cannabinol interfere with this eraser directly, both by binding ferrous iron in the catalytic pocket and by occupying the pocket itself with high binding affinity [88]. It is important to appreciate that cannabinoids not only damage the epigenome generally but also damage the machinery that operates and maintains it [51]. That is to say, the cell’s ability to correct and repair the epigenomic damage is further compromised.

Several detailed epigenome-wide association studies following PCE have been published [51,89]. Interestingly, cardiogenesis was one of the most strongly associated processes in cannabis dependence identified by the in-depth EWAS of the North Carolina group, with an even stronger signal than for brain development [51]. A total of 26 cardiogenic genes identified in their study were impacted by cannabinoid epigenotoxicity. Processes compromised by cannabinoids included morphology of the cardiovasculature, hypoplasia of the trabeculae carnae, number of cardiomyocytes, hypoplasia of the heart chambers, right heart hypoplasia, atrial development, atrial septal morphogenesis, atrial hypoplasia and abnormal atrial morphology, including 127 genes in all [51].

The cardiogenic core gene cassette series was strongly identified in this EWAS, including MEF2 (Myocyte Enhancer Factor 2) NKX2 (NK2 Homeobox 2) GATA (GATA transcription factor family) and TBX (T-box transcription factor family) [51]. This EWAS identified 395 epigenomic hits on this key gene cassette [5,51,84,85,90]. As in other tissues, one of the key cardiac morphogens is sonic hedgehog (SHH) [47]. Genes in the SHH pathway disrupted by cannabis included GLI3 (Gli family zinc finger 3), MEGF8 (multiple EGF-like domains 8), TMEM107 (transmembrane protein 107) and BMP4 (bone morphogenetic protein 4), totaling 583 hits in all [51].

The above introductory commentary thus provides solid evidence of the widespread disruption of key cardiogenic genes, both directly and particularly epigenomically.

### 4.5. Strength of Association

This study met all of the Hill criteria for causality, including particular consistency with external data [5,41,42,43,44,45,46], temporality and the presence of a host of cellular and molecular mechanisms to explain findings.

The use of inverse probability weighting throughout is a powerful pseudo-randomizing statistical technique from which it is appropriate to draw causal inferences within the limitations of the measured predictor covariate matrix. Similarly, the moderate to high E-values strongly support the strength of the association of the C-ASD relationship. Some of the E-values reported are up to 7.77 × 10^−63^, which can be seen when relationships are in fact of a causal nature.

Having said that, other covariates could be considered (listed in the Limitations Section below), which would further constrain the potential contribution of presently unmeasured confounding. As an ecological study, this study is not able to address individual-level exposures.

### 4.6. Generalizability

The size and duration of this dataset, its consistency with other large studies, the logic of its cellular mechanisms, the high E-values and the repeated confirmation of the association in pseudo-randomized inverse probability weighted analyses together indicate that these findings are broadly generalizable [5,41,42,43,44,45,46]. E-values suggested that currently unmeasured confounding would need to be substantial to fully explain the observed associations.

### 4.7. Comparisons to Known Teratogens

Since both tobacco and alcohol are known to have negative effects on fetal development, it is worth considering the impacts of cannabis relative to these known teratogens.

Cigarettes, including ethnic cigarette exposure, appeared in all models in Table 2; however, in each case, it was much less impactful than the effects of cannabis. In the summary VIF table, cannabis accounted for 9% of the total partial variance and tobacco for only 1.7% (Table S5). In the three multivariate survey regression models in Table S6, tobacco exposure was again usually less impactful and less strongly associated with ASDRs than cannabinoid covariates. This pattern was continued in the gam analysis in Table S7. This overall pattern, where cannabis appears to be a much more potent teratogen than tobacco, was continued in large surveys of congenital anomalies at the national and continental levels [5,45,91].

Binge alcohol use appeared in three of the four mixed-effects models shown in Table 2, but its effect was inversely related to ASDRs, which is consistent with a generally falling binge alcohol involvement in the population, generally in the context of a rising ASDR. Binge alcohol use appeared in the final interactive survey regression model, but again, this relationship was inverse (Table S3). When considered by variance decomposition, binge alcohol was twelfth out of fourteen variables considered in Table S4. It was not included amongst the final significant predictive covariates in the survey regressions in Table S6 or the gam regressions in Table S7. This again implies that cannabis is a much more potent teratogen than binge alcohol consumption. Other indices of alcohol exposure may be considered, but as shown in Figures S26–S29, these are likely to yield similar unimpressive results.

Thus, overall, it appears that cannabis is a much more potent cardiac teratogen than tobacco, which, in turn, is a much more potent teratogen than alcohol, as has been previously documented [5,45,91].

### 4.8. Strengths and Limitations

The strengths of this study include its large data size, the use of three different forms of regression, the application of E-values and inverse probability weighting cornerstone techniques of statistical causal inference and the detailed statistical analysis. Its weaknesses include its lack of detailed participant-level data, which is a limitation it shares in common with many epidemiological studies. This ecological study has not considered a number of covariates that are known to be related to congenital anomaly rates, including maternal age, comorbidities, socioeconomic status, education, smoking, alcohol use, access to prenatal care. Such datasets may be explored in subsequent research. Because analyses were conducted at the ecological level, results should be interpreted as population-level associations rather than individual-level causal effects. The present study does not include a space–time analysis nor a heavy ethnocentric focus, which will be addressed in companion papers [92,93]. Earlier single-center retrospective studies have questioned CDC ASDR data integrity based on the finding of 64.3% positive predictive value in juvenile populations [94]. Whilst this issue is of relevance to this work, it does not compromise its major conclusions.

## 5. Conclusions

Data confirm that cannabis is a strong candidate for potentially driving ASDRs, a result amplified by the key biomarker role played by ASD in cannabinoid teratogenicity and cannabinoid genotoxicity more generally. The implications of the exponential cannabinoid genotoxicity dose–response curve are generally overlooked in public health discussions relating to cannabinoid health risks, but based on this and related studies, they are likely very profound indeed. Community genomes and epigenomes are precious but fragile resources that should be carefully protected by strictly limiting cannabinoid exposure of the current generation to protect those of multiple generations to come.

## Figures and Tables

**Table 1:** Social, demographic and substance exposure baseline data.

Covariate	High	Low	∆SMD	*p*-Value
Sample Size (N)	426	1456		
ASD Rate (median [IQR])	88.60 [27.58, 141.95]	44.11 [24.72, 85.50]	0.4042	<0.001
Log (ASD Rate) (median [IQR])	4.48 [3.32, 4.96]	3.79 [3.21, 4.45]	0.4456	<0.001
Numbers Atrial Septal Defect (total)	72,513	334,380	0.5567	<0.001
Births (total)	7,010,509	58,607,743	0.2286	<0.001
Cigarettes, Monthly (median [IQR])	0.24 [0.22, 0.26]	0.25 [0.23, 0.29]	0.4679	<0.001
Alcohol, Monthly (median [IQR])	0.63 [0.59, 0.66]	0.54 [0.48, 0.59]	1.4531	<0.001
Alcoholism (median [IQR])	0.08 [0.07, 0.09]	0.07 [0.06, 0.08]	0.9286	<0.001
Binge Alcohol (median [IQR])	0.27 [0.25, 0.28]	0.24 [0.22, 0.26]	0.8030	<0.001
Cannabis, Monthly (median [IQR])	0.11 [0.08, 0.13]	0.06 [0.05, 0.07]	2.0446	<0.001
Analgesics, Annual (median [IQR])	0.05 [0.04, 0.05]	0.04 [0.04, 0.05]	0.4807	<0.001
Cocaine, Annual (median [IQR])	0.02 [0.02, 0.03]	0.02 [0.01, 0.02]	1.0907	<0.001
∆9THC Content (median [IQR])	13.40 [9.58, 14.60]	12.30 [8.76, 14.10]	0.1858	0.001
Cannabidiol (median [IQR])	0.28 [0.17, 0.46]	0.28 [0.20, 0.46]	0.1923	0.423
Cannabigerol (median [IQR])	0.43 [0.40, 0.47]	0.43 [0.40, 0.46]	0.1657	0.002
Cannabinol (median [IQR])	0.56 [0.38, 0.65]	0.45 [0.37, 0.63]	0.1980	<0.001
Cannabichromene (median [IQR])	0.25 [0.24, 0.27]	0.24 [0.24, 0.26]	0.1179	0.196
Tetrahydrocannabivarin (median [IQR])	0.09 [0.09, 0.10]	0.09 [0.09, 0.10]	0.1514	0.041
∆9THC * Cannabis, Monthly (median [IQR])	1.30 [0.87, 1.81]	0.65 [0.49, 0.90]	1.3607	<0.001
Median Income (median [IQR])	55,928.00 [51,258.50, 60,934.00]	49,047.00 [43,307.00, 56,134.00]	0.7464	<0.001
Ethnic Cigarette Exposure (median [IQR])	0.23 [0.18, 0.27]	0.24 [0.17, 0.29]	0.1087	0.025
Ethnic Analgesic Exposure (median [IQR])	0.08 [0.07, 0.09]	0.07 [0.05, 0.08]	0.3168	<0.001
Ethnic Binge Alcohol (median [IQR])	0.25 [0.22, 0.27]	0.22 [0.18, 0.25]	0.2997	<0.001
Ethnic Cannabis Exposure (median [IQR])	0.10 [0.07, 0.13]	0.06 [0.04, 0.07]	1.1870	<0.001
Ethnic Analgesic Exposure (median [IQR])	0.04 [0.03, 0.05]	0.04 [0.03, 0.05]	0.2767	<0.001
Ethnic Cocaine Exposure (median [IQR])	0.02 [0.02, 0.03]	0.02 [0.01, 0.02]	0.6115	<0.001
Legal Status of Cannabis (%)	4.48 [3.32, 4.96]	3.79 [3.21, 4.45]	0.4456	<0.001
Medical	269 (63.1)	177 (12.2)		<0.001
Legal	64 (15.0)	5 (0.3)		
Illegal	63 (14.8)	1106 (76.0)		
Decriminalized	30 (7.0)	168 (11.5)		
Number Illegal (%)	63 (14.8)	1106 (76.0)	1.5573	<0.001
Number Legal (%)	64 (15.0)	5 (0.3)	0.5734	<0.001

Table Key: IQR—Interquartile Range; SMD—Standardized Mean Difference; *—Interaction.

**Table 2:** Final inverse probability weighted mixed effects models.

Parameter			Model	
Parameter	Estimate (C.I.)	*p*-Value	Metric	Value
Additive in Drugs				
*ASD ~ Cigarettes + Cannabis + Bnge.Alcohol + Analgesics + Cocaine*				
Cigarettes	4.04 (2.25, 5.83)	<0.001	AIC	3305
LM.Cannabis	0.70 (0.52, 0.88)	<0.001	BIC	3344
Analgesics	−0.61 (−0.87, −0.35)	<0.001	LogLik	−1646
Cocaine	−0.13 (−0.25, −0.01)	0.038	S.D.	3.850
Additive in Drugs and Income				
*ASD ~ Cigarettes + Cannabis + Bnge.Alcohol + Analgesics + Cocaine + Med.Income*				
Cigarettes	4.86 (2.99, 6.73)	<0.001	AIC	3294
LM.Cannabis	0.59 (0.39, 0.79)	<0.001	BIC	3338
Bng.Alcohol	−2.75 (−5.10, −0.40)	0.023	LogLik	−1639
Analgesics	−0.53 (−0.79, −0.27)	<0.001	S.D.	3.840
Median.Income	0.75 (0.27, 1.23)	0.002		
Additive in Drugs, Income and Race				
*ASD ~ Cigarettes + Cannabis + Bnge.Alcohol+ Analgesics + Cocaine + Med.Income + Race*				
Cigarettes	4.96 (3.15, 6.77)	<0.001	AIC	3163
LM.Cannabis	0.63 (0.44, 0.82)	<0.001	BIC	3241
Bng.Alcohol	−2.51 (−4.78, −0.24)	<0.001	LogLik	−1568
Analgesics	−0.48 (−0.73, −0.22)	<0.001	S.D.	3.670
Cocaine	−0.13 (−0.25, −0.01)	0.038		
Median.Income	0.75 (0.29, 1.20)	0.001		
Race.Hispanic	0.21 (0.13, 0.29)	<0.001		
Race.NHAIAN	0.42 (0.33, 0.51)	<0.001		
Race.NHBlack	0.47 (0.39, 0.55)	<0.001		
Race.NHWhite	0.15 (0.07, 0.23)	<0.001		
Race.Total	0.24 (0.16, 0.32)	<0.001		
Interactive–Unweighted				
Interactive in Drugs + Income and Race: Cannabis Interaction				
ASD ~ eCigarettes * eCannabis + eBnge.Alcohol + eAnalgesics + eCocaine + Med.Income + Race + Race: Cannabis				
eCigarettes	0.16 (0.07, 0.25)	<0.001	AIC	1805
eCannabis	0.48 (0.37, 0.59)	<0.001	BIC	1910
eBng.Alcohol	−0.10 (−0.18, −0.02)	0.0143	LogLik	−884
eCocaine	−0.11 (−0.15, −0.07)	<0.001	S.D.	0.361
Median.Income	0.08 (0.02, 0.14)	0.0089		
Race.NHAIAN	−0.35 (−0.50, −0.19)	<0.001		
Race.NHAsPI	0.55 (0.13, 0.97)	0.010		
Race.NHBlack	−0.18 (−0.28, −0.07)	0.001		
Race.NHWhite	−0.30 (−0.40, −0.20)	<0.001		
Race.Total	−0.23 (−0.31, −0.14)	2.12 × 10^−7^		
eCigarettes: eCannabis	0.12 (0.03, 0.21)	<0.001		
eCannabis: Race.NHAIAN	−0.21 (−0.37, −0.05)	0.010		
eCannabis: Race.NHAsPI	0.53 (0.18, 0.88)	0.003		
eCannabis: Race.NHBlack	−0.23 (−0.33, −0.12)	<0.001		
eCannabis: Race.NHWhite	−0.25 (−0.37, −0.13)	<0.001		
eCannabis: Race.Total	−0.18 (−0.29, −0.07)	0.001		

Table Key: *—Interaction.

## Data Availability

The original data presented in the study are openly available in the Mendeley database at Mendeley Data, V2, https://doi.org/10.17632/ny2j3msk86.2.

## Supplementary Materials

The following supporting information can be downloaded at: https://www.mdpi.com/article/10.3390/jox16020043/s1, **Supplementary Tables:** Table S1: Twelve periods of analysis and mean ASD rates; Table S2: Cannabis and cannabinoid exposure data; Table S3: Introductory mixed-effects regression final model results; Table S4: Variable importance table from full interactive mixed-effects model; Table S5: Summary variable importance table from full interactive mixed-effects model by main covariate; Table S6: Final survey regression models; Table S7: Final generalized additive model regressions; Table S8: Legal status analysis; Table S9: Pairwise comparison of Cohen’s D by legal status; Table S10: Within- and between-states results from final hybrid mixed-effects regression model; Table S11: Cohen’s D pairwise contrasts from models in Table S10; Table S12: E-values for models and comparisons presented; Table S13: Variance inflation factors for additive and interactive models. **Supplementary Figures:** Figure S1: Log ASD Rate by Ethnicity; Figure S2: ASD Rates by Ethnicity by State x 23 A-M; Figure S3: ASD Rates by Ethnicity by State x 23 M-W; Figure S4: ASD Rates in Nevada by Ethnicity; Figure S5: ASD Rates in New York by Ethnicity; Figure S6: ASD Rates in New York (More Details) by Ethnicity; Figure S7: ASD Rates in Department of Defence by Ethnicity; Figure S8: ASD Rates in Kentucky by Ethnicity; Figure S9: ASD Rates in Michigan by Ethnicity; Figure S10: ASD Rates in Colorado by Ethnicity; Figure S11: ASD Rates in Tennessee by Ethnicity; Figure S12: ASD Rates in Alaska by Ethnicity; Figure S13: ASD Rates in Mississippi by Ethnicity; Figure S14: ASD Rates in Missouri by Ethnicity; Figure S15: ASD Rates in New Mexico by Ethnicity; Figure S16: ASD Rates in Oregon by Ethnicity; Figure S17: ASD Rates in Florida by Ethnicity; Figure S18: ASD Rates in Utah by Ethnicity; Figure S19: ASD Rates in Texas by Ethnicity; Figure S20: ASD Rates in Georgia by Ethnicity; Figure S21: ASD Rates in Maryland by Ethnicity; Figure S22: ASD Rates in Minnesota by Ethnicity; Figure S23: ASD Rates in Iowa by Ethnicity; Figure S24: ASD Rates in South Carolina by Ethnicity; Figure S25: ASD Rates in New Jersey by Ethnicity; Figure S26: Rates of Substance Exposure in USA; Figure S27: ASD Rates by Selected Substances, Loess Lines; Figure S28: ASD Rates by Selected Substances, Regression Lines; Figure S29: Log (ASD Rates) by Selected Substances, Regression Lines; Figure S30: Cannabinoid Exposure Trends Across USA; Figure S31: Slopes ASD by Time v ASD by Cannabis Regression lines; Figure S32: Variable Importance Plot; Figure S33: Trends in Cannabis Legal Status Across USA States.

## Abbreviations

The following abbreviations are used in this manuscript:

AIC	Akaike Information Criterion
alcmon	Alcohol use in the past year
anlyr	Analgesic misuse in the past year
ASD	Atrial septal defect, secundum type
ASDR	Atrial septal defect rate
audyr	Alcohol use disorder in the past year
BIC	Bayesian Information Criterion
bngalc	Binge alcohol use in the past year
BMP	Bone morphogenetic proteins
CBG	Cannabigerol
CENPN	Centrosomal Protein N
C.I.	Confidence Interval
cigmon	Cigarette use in the past month
COUP-TF II	Chicken Ovalbumin Upstream Promoter Transcription Factor II
CRAN	Central R Archive Network
cocyr	Cocaine use in the past year
EDF	Estimated degrees of freedom
EWAS	Epigenome-wide association study
E-Value	Expected value
Δ9THC	Δ9-tetrahydrocannabinol
GATA	GATA transcription factor family
Hispanic	Hispanic
IQR	Interquartile Range
logLik	Log Likelihood ratio at model maximization
GLI3	Gli family zinc finger 3
MEF2	Myocyte Enhancer Factor 2
MEGF8	Multiple EGF-like domains 8
mgcv	Mixed Gam Computation Vehicle
mrjmon	Cannabis use in the past month
NBDPN	National Birth Defect Prevention Network
NHWhite	Non-Hispanic White
NHBlack	Non-Hispanic Black
NHAIAN	Non-Hispanic American Indian Alaskan Native
NHAsPI	Non-Hispanic Asian/Pacific Islander
NKX2	NK2 Homeobox 2
NSDUH	National Survey of Drug Use and Health
Overall	Overall or total of all races combined
PCE	Prenatal Cannabis Exposure
SAMHSA	Substance Abuse and Mental Health Services Administration
SD	Standard deviation
SEM	Standard error of the mean
SHH	Sonic hedgehog
TBX	T-box transcription factor family
TMEM107	Transmembrane protein 107
VEGF	Vascular Endothelial Growth Factor
VIF	Variance inflation factor
