# Supplementary material for: Impact of Cannabis and Cannabis Legalization on US Atrial Septal Defect Rates
Source: J Xenobiot. 2026 Mar 1;16(2):43. doi: 10.3390/jox16020043 (PMC13010746; doi:10.3390/jox16020043)

Supplementary Materials: Impact of Cannabis and Cannabis Legalization on US Atrial Septal Defect Rates

Albert Stuart Reece and Gary Kenneth Hulse

Supplementary Tables:

Table S1.: 12 Periods of Analysis and mean ASD Rates
Table S2.: Cannabis and Cannabinoid Exposure Data
Table S3.: Introductory Mixed Effects Regression Final Model Results
Table S4.: Variable Importance Table from Full Interactive Mixed Effects Model
Table S5.: Summary Variable Importance Table from Full Interactive Mixed Effects Model by main covariate
Table S6.: Final Survey regression models
Table S7.: Final Generalized Additive Model Regressions
Table S8.: Legal status analysis
Table S9.: Pairwise comparison of Cohen's D by legal status
Table S10.: Within- and Between- States results from final hybrid mixed effects regression model.
Table S11.: Cohen's D Pairwise Contrasts from models of Table S 10
Table S12.: E-Values from models and comparisons presented.
Table S13.: Variance Inflation Factors (VIF's) from Table 2

Supplementary Figures

Figure S1 – Log ASD Rate by Ethnicity
Figure S2 - ASD Rates by Ethnicity by State x 23 A-M
Figure S3 - ASD Rates by Ethnicity by State x 23 M-W
Figure S4 - ASD Rates in Nevada by Ethnicity
Figure S5 - ASD Rates in New York by Ethnicity
Figure S6 - ASD Rates in New York (more details) by Ethnicity
Figure S7 - ASD Rates in Department of Defence by Ethnicity
Figure S8 - ASD Rates in Kentucky by Ethnicity
Figure S9 - ASD Rates in Michigan by Ethnicity
Figure S10 - ASD Rates in Colorado by Ethnicity
Figure S11 - ASD Rates in Tennessee by Ethnicity
Figure S12 - ASD Rates in Alaska by Ethnicity
Figure S13 - ASD Rates in Mississippi by Ethnicity
Figure S14 - ASD Rates in Missouri by Ethnicity
Figure S15 - ASD Rates in New Mexico by Ethnicity
Figure S16 - ASD Rates in Oregon by Ethnicity
Figure S17 - ASD Rates in Florida by Ethnicity
Figure S18 - ASD Rates in Utah by Ethnicity
Figure S19 - ASD Rates in Texas by Ethnicity
Figure S20 - ASD Rates in Georgia by Ethnicity
Figure S21 - ASD Rates in Maryland by Ethnicity
Figure S22 - ASD Rates in Minnesota by Ethnicity
Figure S23 - ASD Rates in Iowa by Ethnicity
Figure S24 - ASD Rates in South Carolina by Ethnicity
Figure S25 - ASD Rates in New Jersey by Ethnicity
Figure S26 – Rates of Substance Exposure in USA
Figure S27 - ASD Rates by Selected Substances, Loess Lines
Figure S28 - ASD Rates by Selected Substances, Regression Lines
Figure S29 – Log (ASD Rates) by Selected Substances, Regression Lines
Figure S30 – Cannabinoid Exposure Trends Across USA
Figure S31 – Slopes ASD by Time v ASD by Cannabis Regression lines
Figure S32 – Variable Importance Plot
Figure S33 – Trends in Cannabis Legal Status Across USA States

Table S1.: 12 Periods of Analysis and Mean ASD Rates

Period	Year	Group	Mean ASD Rate
1989-1990	1990	1	27.4
1995-1999	1997	2	33.9
2003-2007	2005	3	51.2
2004-2008	2006	4	59.2
2005-2009	2007	5	58.2
2007-2011	2009	6	56.3
2008-2012	2010	7	67.1
2009-2012	2011	8	69.3
2010-2014	2012	9	75.9
2011-2015	2013	10	86.5
2012-2016	2014	11	90.3
2016-2020	2018	12	116.0

Table S2.: Cannabis and Cannabinoid Exposure Data

Year	Δ9THC	CBD	CBC	CBN	CBG	THCV	High Potency	Monthly Cannabis	THC * HiPotency * Monthly Use
**2007**	9.58	0.46	0.24	0.31	0.44	0.1	-	5.89	-
**2008**	8.9	0.41	0.25	0.32	0.37	0.1	-	6.33	-
**2009**	8.3	0.39	0.24	0.37	0.33	0.1	28.16	6.71	1,567.78
**2010**	10	0.28	0.25	0.43	0.34	0.08	33.52	6.87	2,301.18
**2011**	12.3	0.22	0.25	0.45	0.42	0.09	40.31	6.81	3,374.11
**2012**	14.1	0.2	0.24	0.56	0.43	0.09	47.00	7.09	4,697.88
**2013**	13.4	0.17	0.27	0.63	0.47	0.1	44.91	7.43	4,469.76
**2014**	14.6	0.15	0.23	0.65	0.46	0.09	43.53	8.04	5,109.03
**2015**	13.4	0.18	0.22	0.75	0.47	0.08	33.97	8.45	3,847.70
**2016**	13.2	0.19	0.23	0.73	0.46	0.08	41.15	8.79	4,775.64
**2017**	14.13	0.56	0.28	0.62	0.54	0.09	58.82	9.51	7,903.35
**2018**	14.88	0.71	0.29	0.7	0.58	0.19	68.27	10.16	10,320.50

Table S3.: Introductory Mixed Effects Regression Final Model Results

Parameter			Model	
**Parameter**	**Estimate (C.I.)**	**P-Value**	**Metric**	**Value**
**Simple**			AIC	3,327
ASD ~ LM.Cannabis			BIC	3,349
LM.Cannabis	0.62 (0.48, 0.76)	1.41E-17	LogLik	-1,659
			S.D.	3.870
ASD ~ Δ9THC			AIC	3,356
Δ9THC	0.39 (0.28, 0.50)	3.36E-11	BIC	3,378
			LogLik	-1,674
			S.D.	3.900
ASD ~ LM.Cannabis + Δ9THC			AIC	3,328
Δ9THC	0.14 (0.00, 0.28)	0.0454	BIC	3,356
LM.Cannabis	0.52 (0.34, 0.69)	9.91E-09	LogLik	-1,659
			S.D.	3.870
Interactive in Cannabis				
*ASD ~ LM.Cannabis * Δ9THC*			AIC	3,331
LM.Cannabis	0.65 (0.51, 0.80)	1.91E-18	BIC	3,365
Δ9THC: LM.Cannabis	-0.05 (-0.10, 0.00)	0.0422	LogLik	-1,660
			S.D.	3.870
Interactive in Cannabinoids			AIC	3,327
*ASD ~ LM.Cannabis * Δ9THC * CBG*			BIC	3,355
LM.Cannabis	0.52 (0.35, 0.69)	4.06E-09	LogLik	-1,659
Cannabigerol	0.21 (0.01, 0.41)	0.0414	S.D.	3.870
Additive in Ethnic Drugs, Income and Race				
ASD ~ eCigarettes + eCannabis + eBnge.Alcohol + eAnalgesics + eCocaine + Med.Income + Race				
eCigarettes	-0.55 (-1.09, -0.01)	4.77E-02	AIC	33,593
eCannabis	9.62 (8.04, 11.20)	1.26E-31	BIC	33,660
eAnalgesics	19.4 (16.77, 22.03)	6.52E-45	LogLik	-16,785
sCocaine	-27.8 (-32.25, -23.35)	2.77E-33	S.D.	1,484
Race.NHBlack	0.35 (0.24, 0.46)	1.49E-09		
Race.NHWhite	-0.66 (-0.78, -0.55)	3.92E-28		
Race.Total	-0.23 (-0.33, -0.13)	8.46E-06		

Interactive				
Interactive in Drugs + Income and Race : Cannabis Interaction				
*ASD ~ eCigarettes * eCannabis + eBnge.Alcohol + eAnalgesics + eCocaine + Med.Income + Race + Race: Cannabis*				
eCigarettes	-18.9 (-20.41, -17.39)	8.53E-115	AIC	32,860
eCannabis	-15.8 (-24.4, -7.2)	0.0003	BIC	32,971
eAnalgesics	13.5 (10.09, 16.91)	1.22E-14	LogLik	-16,410
eBng.Alcohol	-1.76 (-2.86, -0.66)	0.0018	S.D.	1,223
eCocaine	-24.5 (-28.36, -20.64)	3.50E-34		
Median.Income	0.53 (0.25, 0.81)	2.38E-04		
Race.Hispanic	1.96 (1.70, 2.22)	1.55E-46		
Race.NHAIAN	3.98 (3.47, 4.49)	1.11E-49		
Race.NHBlack	3.45 (3.17, 3.73)	2.06E-109		
Race.NHWhite	2.74 (2.42, 3.06)	7.63E-60		
Race.Total	2.57 (2.28, 2.86)	1.16E-62		
eCannabis: eCigarettes	290 (267.66, 312.34)	1.72E-122		
eCannabis: RaceHispanic	-15.60 (-22.21, -8.99)	3.81E-06		
eCannabis: Race.NHAIAN	-50.4 (-58.87, -41.93)	1.84E-30		
eCannabis: Race.NHBlack	-35.6 (-42.58, -28.62)	5.65E-23		
eCannabis: Race.NHWhite	-38.5 (-45.54, -31.46)	4.33E-26		
eCannabis: Race.Total	-29.8 (-36.74, -22.86)	7.16E-17		

Table S4.: Variable Importance Table S from Full Interactive Mixed Effects Model

Group	Term	Estimate	CI_lower	CI_upper
Cannabis	Eth.Cannabis	0.0234	0.0080	0.0536
Cannabis	Eth.Cannabis + Eth.Cigarettes:Eth.Cannabis + Eth.Cannabis:Race	0.0193	0.0039	0.0489
Cannabis	Eth.Cigarettes:Eth.Cannabis + Eth.Cannabis:Race	0.0155	0.0001	0.0443
Cannabis	Eth.Cannabis:Race	0.0149	0.0000	0.0436
Cannabis	Eth.Cannabis + Eth.Cannabis:Race	0.0111	0.0000	0.0396
Race	Race	0.0121	0.0000	0.0396
Ciga-rettes	Eth.Cigarettes + Eth.Cigarettes:Eth.Cannabis	0.0108	0.0000	0.0371
Cannabis	Eth.Cigarettes:Eth.Cannabis	0.0030	0.0000	0.0313
Cannabis	Eth.Cannabis + Eth.Cigarettes:Eth.Cannabis	0.0030	0.0000	0.0313
Ciga-rettes	Eth.Cigarettes	0.0031	0.0000	0.0292
Ciga-rettes	Eth.Cigarettes:Eth.Cannabis	0.0030	0.0000	0.0292
Bngalc	Eth.Binge.Alcohol	0.0000	0.0000	0.0256
MHY	Median.Income	0.0000	0.0000	0.0237
Cocaine	Eth.Cocaine	0.0000	0.0000	0.0197

Table S5.: Summary Variable Importance Table from Full Interactive Mixed Effects Model by main covariate

Group	total_partial_R2	max_CI_upper	n_terms
Cannabis	0.0903	0.0536	7
Cigarettes	0.0169	0.0371	3
Race	0.0121	0.0396	1
Binge Alcohol	0	0.0256	1
Median Income	0	0.0237	1
Cocaine	0	0.0197	1

Table S6.: Final Survey regression models

Parameter			Model	
**Parameter**	**Estimate (C.I.)**	**P-Value**	**Metric**	**Value**
**Simple**				
ASD ~ LM.Cannabis			AIC	5744
LM.Cannabis	0.5 (0.38, 0.62)	2.01E-17	BIC	2344
			Deviance	2329
ASD ~ Δ9THC		4.46E-20	AIC	5760
Δ9THC	0.58 (0.36, 0.79)	1.68E-07	BIC	2363
			Deviance	2348
ASD ~mrjmon + Δ9THC				
LM.Cannabis	0.41 (0.29, 0.53)	3.25E-11	AIC	5739
Δ9THC	0.33 (0.1, 0.56)	4.91E-03	BIC	2343
			Deviance	2320
ASD ~mrjmon x Δ9THC			AIC	5737
Δ9THC x LM.Cannabis	0.38 (0.29, 0.47)	1.93E-15	BIC	2335
			Deviance	2320
ASD ~mrjmon * Δ9THC				
LM.Cannabis	-2.26 (-3.77, -0.75)	0.0033	AIC	5731
Δ9THC	3.24 (1.57, 4.91)	0.0001	BIC	2339
LM.Cannabis: Δ9THC	1.07 (0.44, 1.7)	0.0008	Deviance	2309
ASD ~ mrjmon * d9THC * CBG				
Δ9THC	1.5 (0.83, 2.17)	1.29E-05	AIC	5733
Cannabigerol	2.33 (0.74, 3.92)	0.0041	BIC	2346
LM.Cannabis: Δ9THC	0.44 (0.22, 0.67)	0.0001	Deviance	2309
LM.Cannabis: Cannabigerol	0.86 (0.24, 1.48)	0.0064		
**Additive**				
ASD ~Cigarettes + mrjmon x d9THC + bngalc + anlyr + cocyr + Med.Income + Race				
Cigarettes	4.97 (3.72, 6.22)	9.04E-15	AIC	5391
Δ9THC x LM.Cannabis	0.84 (0.75, 0.93)	2.76E-68	BIC	1985
Median.Income	-1.72 (-2.02, -1.42)	1.37E-28	Deviance	1917
Race.NHAIAN	0.6 (0.44, 0.76)	2.51E-13		

Race.NHBlack	0.42 (0.25, 0.59)	2.02E-06		
**Interactive**				
ASD ~Cigarettes + mrjmon * d9THC * CBG + bngalc + anlyr + cocyr + Med.Income + Race				
Cigarettes	5.16 (3.87, 6.45)	6.69E-15	AIC	5362
LM.Cannabis	-2.29 (-3.76, -0.82)	2.18E-03	BIC	1986
Δ9THC	6.52 (4.4, 8.64)	1.88E-09	Deviance	1880
Cannabigerol	-6.57 (-10.47, -2.67)	9.97E-04		
Analgesics	0.42 (0.16, 0.68)	1.44E-03		
Median.Income	-1.62 (-1.93, -1.31)	9.68E-25		
Race.NHAIAN	0.6 (0.44, 0.76)	2.84E-13		
Race.NHBlack	0.42 (0.25, 0.59)	1.60E-06		
Race.NHWhite	0.11 (-0.05, 0.27)	0.1540		
Race.Total	0.2 (0.04, 0.36)	0.0131		
LM.Cannabis: Δ9THC	1.24 (0.64, 1.84)	5.63E-05		
Δ9THC: Cannabigerol	2.88 (1.28, 4.48)	0.0004		
**Interactive Including Race: Cannabis**				
ASD ~ Cigarettes + mrjmon * d9THC * CBG + bngalc + anlyr + cocyr + Med.Income + Race + Race: Cannabis				
Cigarettes	117 (17.43, 216.57)	0.0216	AIC	5351
LM.Cannabis	-15.5 (-23.99, -7.01)	0.0003	BIC	2008
Δ9THC	14.5 (4.99, 24.01)	0.0028	Deviance	1858
Cannabigerol	0.6 (0.14, 1.06)	0.0100		
Median.Income	-1.85 (-2.16, -1.54)	6.73E-31		
Race.Hispanic	1.53 (0.17, 2.89)	0.0276		
Race.NHAIAN	1.4 (0.05, 2.75)	0.0413		
Cigarettes: LM.Cannabis	49 (13.92, 84.08)	0.0064		
LM.Cannabis: Δ9THC	6.07 (2.66, 9.48)	0.0005		
LM.Cannabis: Race.Hispanic	0.51 (0.01, 1.01)	0.0466		
Cigarettes: LM.Cannabis: Δ9THC	-17.6 (-31.85, -3.35)	0.0156		

Table S7.: Final Generalized Additive Model Regressions

Term	Coefficients				Model	
	EDF	Ref.DF	Statistic	P-Value	Index	Value
**Cannabis Models**					DF	164
ASD ~ Ethnic.Cannabis.Exp					LogLik	-9418
s(eCannabis)	1	1	7.93	0.0049	AIC	19164
s(Year,State)	162	325	1968	0.0000	Dev.Expl.	89.6%
ASD ~ THC.Exp					DF	76.8
s(d9THC)	4.98	5	155	0.0000	LogLik	-9433
s(Year,State)	70.8	323	1904	0.0000	AIC	19020
					Dev.Expl.	92.3%
ASD ~ Ethnic.Cannabis.Exp + THC.Exp					DF	77.2
s(eCannabis)	1	1	8.46	0.0036	LogLik	-9383
s(Δ9THC)	8.82	8.92	256	0.0000	AIC	18921
s(Year,State)	66.4	323	1784	0.0000	Dev.Expl.	92.3%
ASD ~ teIR (Ethnic.Cannabis.Exp : THC)					DF	79.8
teIR (eCannabis, Δ9THC))	8.25	8.71	42.2	3.83E-07	LogLik	-9474
s(Year,State)	70.5	323	1818	0.0000	AIC	19107
					Dev.Expl.	85.2%
ASD ~ teIR (Ethnic.Cannabis.Exp : THC : Cigarettes)					DF	167
teIR (eCannabis : THC : Cigarettes)	3	3	7.91	0.0480	LogLik	-9417
s(Year,State)	163	325	1930	0.0000	AIC	19168
					Dev.Expl.	89.7%
**Full Model - Time Interactions**						
ASD ~ Cigarettes + Cannabis + Bng.Alcohol + Analgesics + Cocaine + Med.Income + Race + tiIR (Cigarettes: Time) + tiIR (Cannabis: Time)						
**Parametric Terms**					DF	79.8
	**Estimate**	**Std.Error**	**z-Value**	**P-Value**	LogLik	-9484
Race.Hispanic	-0.1938	0.0861	-2.251	0.0244	AIC	19128
Race.NHAIAN	0.1923	0.0979	1.964	0.0495	Dev.Expl.	84.4%
**Smoothened Terms**						
	**EDF**	**Ref.DF**	**Chi.Squ**	**P-Value**		
s(Cannabis)	1.000	1.001	11.281	0.0008		

tiIR (Cigarettes : Time)	1.000	1.000	9.069	0.0026		
tiIR (Cannabis : Time)	1.502	1.817	9.895	0.0087		
s(Med.Income)	7.243	8.064	21.757	0.0051		
s(Year,State)	63.060	323.000	1595.549	0.0000		
**Full Model - Cannabis: Ethnicity Interaction**						
ASD ~ Cigarettes + Cannabis + Bng.Alcohol + Analgesics + Cocaine + Med.Income + Race + tiIR(Cannabis: Race)						
**Parametric Terms**						
	**Estimate**	**Std.Error**	**z-Value**	**P-Value**	DF	56.6
Hispanic	-0.2329	0.1061	-2.195	0.0282	LogLik	-9490
					AIC	19094
**Smoothened Terms**					Dev.Expl.	83.9%
	**EDF**	**Ref.DF**	**Chi.Squ**	**P-Value**		
s(eCannabisj	2.33	3.01	29.5	2.07E-06		
ti(Year, eCannabis)	2.31	2.73	7.78	0.0256		
s(Median.Income)	7.22	8.1	26.1	0.0010		
s(eCocaine)	1.07	1.14	20.8	2.16E-05		
s(State)	37.7	41	1550	0.0000		

Table Key: * - Interaction; eCannabis – Ethnic Cannabis

Table S8.: Legal status analysis

Data		Tests		
**Legal Status**	**ASD Rates (Mean, C.I.)**	**Test**	**Value**	**P-Value**
Legal Status				
Illegal	67.82 (63.8, 71.84)			
Medical / Decriminalized	94.74 (86.37, 103.11)			
Legal	190.19 (141.33, 239.05)	Chi.Squ.Trend	2,724	0.0023
Legal v Illegal		Student's t	9.07	1.51E-13
Legal Status - Dichotomized				
Not Legal	74.28 (70.6, 77.96)	Student's t	7.47	1.27E-07
Legal	178.15 (131.68, 224.62)	Chi.Squ.	22,194	Zero
		O.R.	1.82 (1.81, 1.84)	
		R.R.	1.79 (1.77, 1.80)	
		AFE	44.92% (44.48%, 45.35%)	
		E-Values	3.04, 3.02	
		NNH	1 in 48 (48, 49)	

Table S9.: Pairwise Comparison of Cohen's D by Legal Status

Contrast	Cohens D (C.I.)	Adj.P.Value
Decriminalized - Illegal	0.20 (0.31, 0.70)	0.8642
Legal - Illegal	1.29 (0.96, 1.62)	2.23E-11
Medical - Illegal	0.50 (0.31, 0.70)	1.98E-06
Legal - Decriminalized	1.10 (0.54, 1.66)	5.59E-04
Medical - Decriminalized	0.31 (0.22, 0.84)	0.6417
Legal - Medical	0.79 (0.51, 1.07)	1.38E-07

Table S10.: Within- and Between- States Results from Final Hybrid Mixed Effects Regression Model.

Parameter			Model	
Parameter	Estimate (C.I.)	P-Value	Parameter	Value
Bivariate Model				
ASD ~ Legal Status				
StatusLegal	0.50 (0.38, 0.63)	4.23E-15	AIC	2002
StatusDecriminalized	0.08 (-0.12, 0.27)	0.4350	BIC	2035
StatusMedical	0.20 (0.12, 0.27)	3.19E-07	LogLik	-995
			S.D.	0.389
			Conditional Variance	0.692
			Marginal Variance	0.028
			State S.D.	0.572
			Residual S.D.	0.389
Additive Model				
ASD ~ Within_Cannabis + Between_Cannabis + Legal Status				
Between_Cannabis	0.14 (0.11, 0.167)	2.56E-19	AIC	1884
Within_Cannabis	0.31 (0.23, 0.386)	8.25E-14	BIC	1928
StatusLegal	0.16 (0.01, 0.309)	0.0351	LogLik	-934
StatusDecriminalized	-0.04 (-0.23, 0.146)	0.6680	S.D.	0.376
StatusMedical	0.05 (-0.04, 0.127)	0.2740	Conditional Variance	0.713
			Marginal Variance	0.046
			State S.D.	0.573
			Residual S.D.	0.376
Interactive Model				
ASD ~ Within_Cannabis * Between_Cannabis * Legal Status				
Between_Cannabis	0.12 (0.08, 0.16)	5.90E-08	AIC	1868
Within_Cannabis	0.29 (0.18, 0.4)	1.07E-07	BIC	1967
StatusLegal	0.03 (-0.2, 0.25)	0.8130	LogLik	-916
StatusDecriminalized	0.00 (-0.19, 0.2)	0.9790	S.D.	0.371
StatusMedical	0.13 (0.03, 0.22)	0.0089	Conditional Variance	0.719
Between_Cannabis: Within_Cannabis	-0.26 (-0.53, 0.01)	0.0604	Marginal Variance	0.068
Between_Cannabis: StatusLegal	0.19 (0.01, 0.37)	0.0398	State S.D.	0.564
Between_Cannabis: StatusDecriminalized	0.11 (0.00, 0.22)	0.0408	Residual S.D.	0.371
Between_Cannabis: StatusMedical	0.04 (-0.03, 0.10)	0.2690		
Within_Cannabis: StatusLegal	0.83 (0.45, 1.20)	0.0000		
Within_Cannabis: StatusDecriminalized	0.65 (0.29, 1.01)	0.0004		
Within_Cannabis: StatusMedical	-0.19 (-0.38, 0.01)	0.0589		
Between_Cannabis: Within_Cannabis: StatusLegal	-0.65 (-1.13, -0.16)	0.0094		
Between_Cannabis: Within_Cannabis: StatusDecriminalized	-1.14 (-1.95, -0.34)	0.0054		
Between_Cannabis: Within_Cannabis: StatusMedical	0.45 (0.11, 0.80)	0.0100		

Table S11.: Cohen's D Pairwise Contrasts from models of Table S 10

Pairwise Contrast	Cohen's D (C.I.)
Bivariate Model	
Legal - Illegal	1.29 (0.96, 1.62)
Decriminalized - Illegal	0.20 (-0.31, 0.070)
Medical - Illegal	0.50 (0.31, 0.70)
Legal - Decriminalized	1.10 (0.54, 1.66)
Legal - Medical	0.79 (0.51, 1.07)
Medical - Decriminalized	0.31 (- 0.22, 0.84)
Additive Model	
Legal - Illegal	0.43 (0.02, 0.83)
Decriminalized - Illegal	-0.11 (-0.62, 0.40)
Illegal - Medical	0.12 (-0.10, 0.34)
Legal - Decriminalized	0.54 (-0.04, 1.11)
Legal - Medical	0.31 (-0.01, 0.62)
Medical - Decriminalized	0.23 (-0.30, 0.76)
Interactive Model	
Legal - Illegal	0.06 (-0.57, 0.69)
Decriminalized - Illegal	0.00 (0.55, -0.55)
Illegal - Medical	0.33 (0.08, 0.59)
Legal - Decriminalized	0.06 (-0.72, 0.84)
Legal - Medical	-0.28 (-0.86, 0.31)
Medical - Decriminalized	0.34 (-0.24, 0.91)

Table S12.: E-Values from models and comparisons presented

Parameter	E-Value Estimate and 97.5% Lower Bounds
**Mixed Effects Models**	
eCannabis	7.77E63, 1.97E50
cigmon : LM.Cannabis	723.65, 102.63
LM.Cannabis	118.62, 38.39
LM.Cannabis	117.00, 24.69
Cigarettes : LM.Cannabis	15.61, 7.22
Cannabigerol	3.60, 2.85
Δ9THC	4.12, 3.78
Δ9THC	3.39, 2.80
Δ9THC : LM.Cannabis	3.60, 3.44
LM.Cannabis	4.88, 4.42
LM.Cannabis	4.53, 3.99
LM.Cannabis	4.54, 4.10
LM.Cannabis	4.54, 4.01
LM.Cannabis	4.54, 3.09
LM.Cannabis	5.14, 4.52
LM.Cannabis	4.78, 4.15
LM.Cannabis	5.00, 4.34
LM.Cannabis : Race.Hispanic	4.10, 3.33
LM.Cannabis : Race.NHAIAN	3.97, 3.15
eCannabis	3239.32, 10.65
**Survey Models**	
Δ9THC	1356.66, 162.4
Δ9THC	50.56, 9.08
Δ9THC	8.43, 4.02
Δ9THC	2.97, 2.22
LM.Cannabis	2.68, 2.28
Δ9THC	2.13, 1.45
Cannabigerol	20.04, 3.61
LM.Cannabis	24.82, 16.97
LM.Cannabis	2.38, 2.01
Δ9THC : Cannabigerol	35.12, 6.65
Δ9THC x LM.Cannabis	2.28, 2.01
Δ9THC x LM.Cannabis	4.06, 3.65
LM.Cannabis : Cannabigerol	4.16, 1.86
LM.Cannabis : Δ9THC	5.28, 2.48

LM.Cannabis : Δ9THC	2.48, 1.8
LM.Cannabis : Δ9THC	6.37, 3.2
eCannabis	2.14E+09, 5.74E+06
**Polynomial Models**	
eCannabis	4.90E+39, 3.00E+06
eCannabis	2.77E+03, 673.22

Table S13.: Variance Inflation Factors (VIF's) from Table 2

Table S 13A – VIF's for Additive Models

Term	VIF	VIF 95% CI	adj. VIF	Tolerance	Tolerance 95% CI
**Model 1 - Additive in Drugs**					
Cigarettes	2.51	[2.34, 2.70]	1.58	0.4	[0.37, 0.43]
LM.Cannabis	1.73	[1.62, 1.85]	1.31	0.58	[0.54, 0.62]
Analgesics	2.08	[1.95, 2.23]	1.44	0.48	[0.45, 0.51]
Cocaine	1.13	[1.08, 1.20]	1.06	0.88	[0.83, 0.92]
**Model 2 - Additive in Drugs & Income**					
Cigarettes	2.74	[2.55, 2.96]	1.66	0.36	[0.34, 0.39]
LM.Cannabis	1.99	[1.87, 2.14]	1.41	0.5	[0.47, 0.54]
Bng.Alcohol	1.12	[1.08, 1.19]	1.06	0.89	[0.84, 0.93]
Analgesics	2.18	[2.04, 2.34]	1.48	0.46	[0.43, 0.49]
log(MHY)	2.24	[2.09, 2.41]	1.5	0.45	[0.42, 0.48]
**Model 3 - Additive in Drugs, Income and Race**					
Cigarettes	2.79	[2.60, 3.01]	1.67	0.36	[0.33, 0.39]
LM.Cannabis	2.07	[1.94, 2.22]	1.44	0.48	[0.45, 0.52]
Bng.Alcohol	1.13	[1.08, 1.20]	1.06	0.89	[0.83, 0.92]
Analgesics	2.24	[2.09, 2.41]	1.50	0.45	[0.42, 0.48]
Cocaine	1.14	[1.09, 1.21]	1.07	0.88	[0.83, 0.92]
Median.Income	2.24	[2.09, 2.41]	1.50	0.45	[0.42, 0.48]
Race	1.00	[1.00, Inf]	1.00	1.00	[0.00, 1.00]

Table S13B – VIF's for Interactive Model 4

Model 4 – Interactive in Drugs and Race

Term	GVIF	DF	GVIF^(1/(2*DF))
eCigarettes	598.71	3	2.90
eCannabis	15.73	13	1.11
eBng.Alcohol	5.74	1	2.40
eCocaine	2.49	1	1.58
Median.Income	2.31	1	1.52
Race	125.90	11	1.25

Figure S1.: Log ASD Rate by Ethnicity

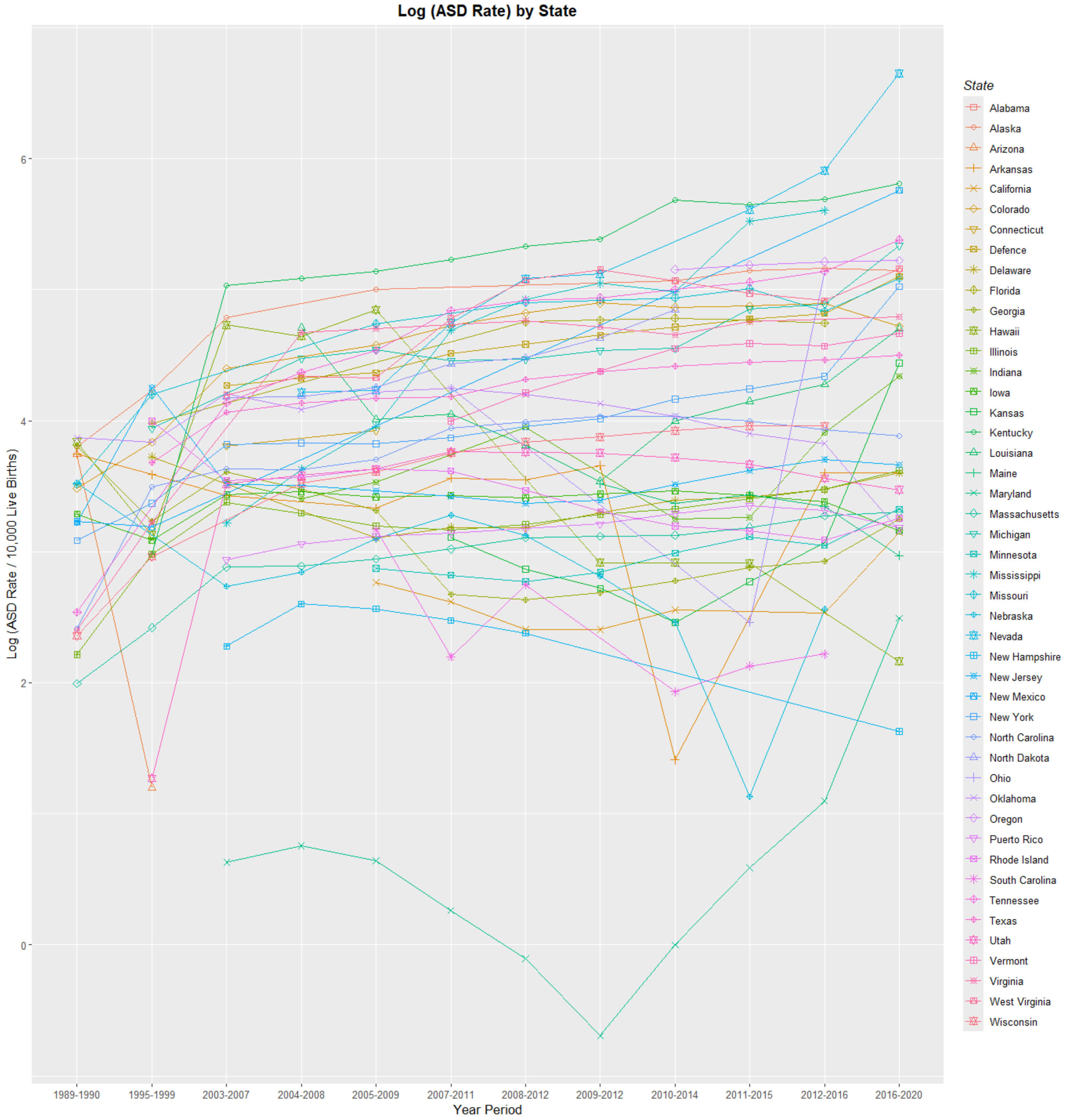

Figure S2.: ASD Rates by Ethnicity by State x 23 A-M

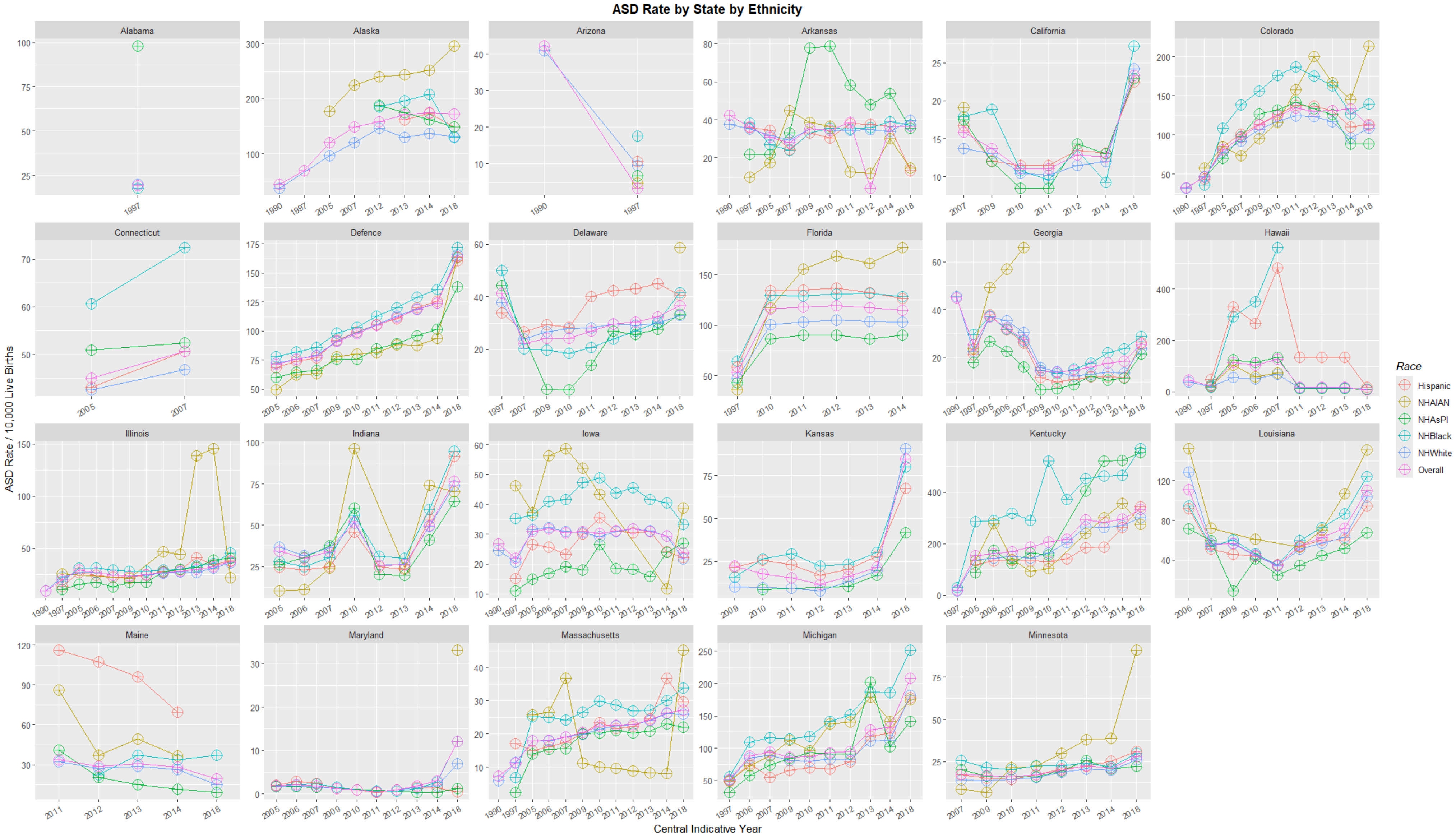

Figure S3.: ASD Rates by Ethnicity by State x 23 M-W

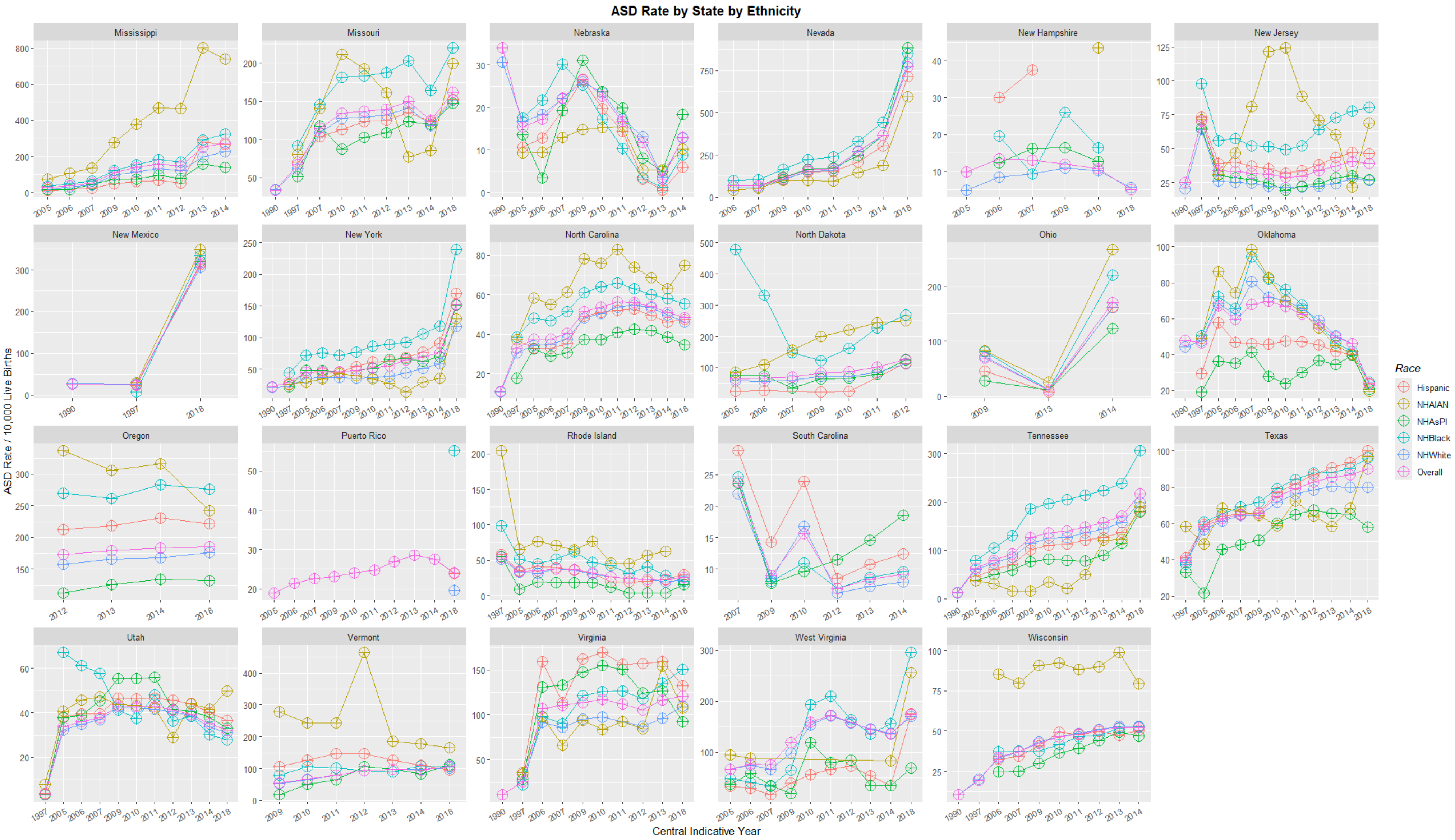
ASD Rate by State by Ethnicity
Mississippi
Missouri
Nebraska
Nevada
New Hampshire
New Jersey
New Mexico
New York
North Carolina
North Dakota
Ohio
Oklahoma
Oregon
Puerto Rico
Rhode Island
South Carolina
Tennessee
Texas
Utah
Vermont
Virginia
West Virginia
Wisconsin
ASD Rate / 10,000 Live Births
Central Indicative Year
Race
Hispanic
NHAIAN
NHAsPI
NHBlack
NHWhite
Overall

Figure S4.: ASD Rates in Nevada by Ethnicity

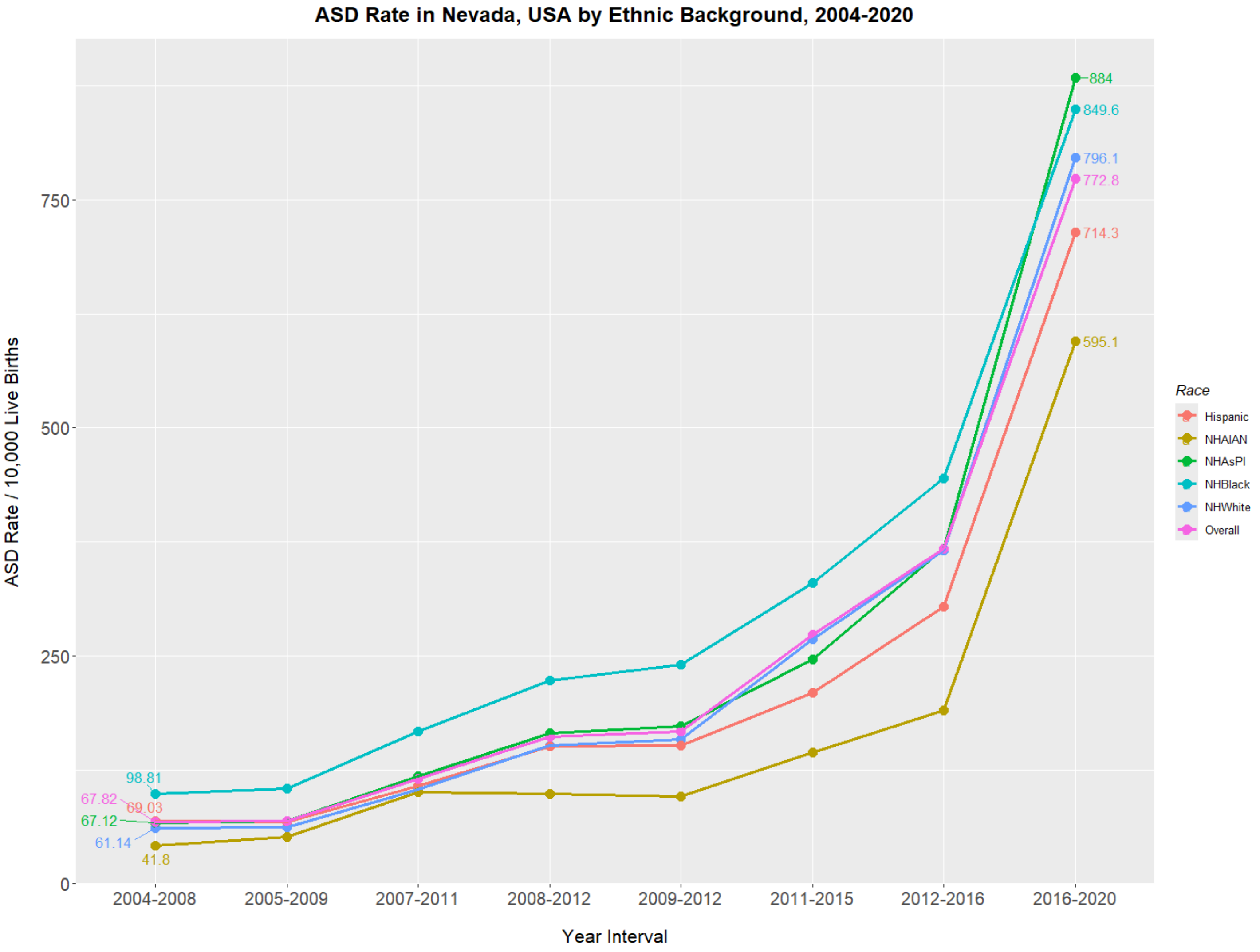

Figure S5.: ASD Rates in New York by Ethnicity

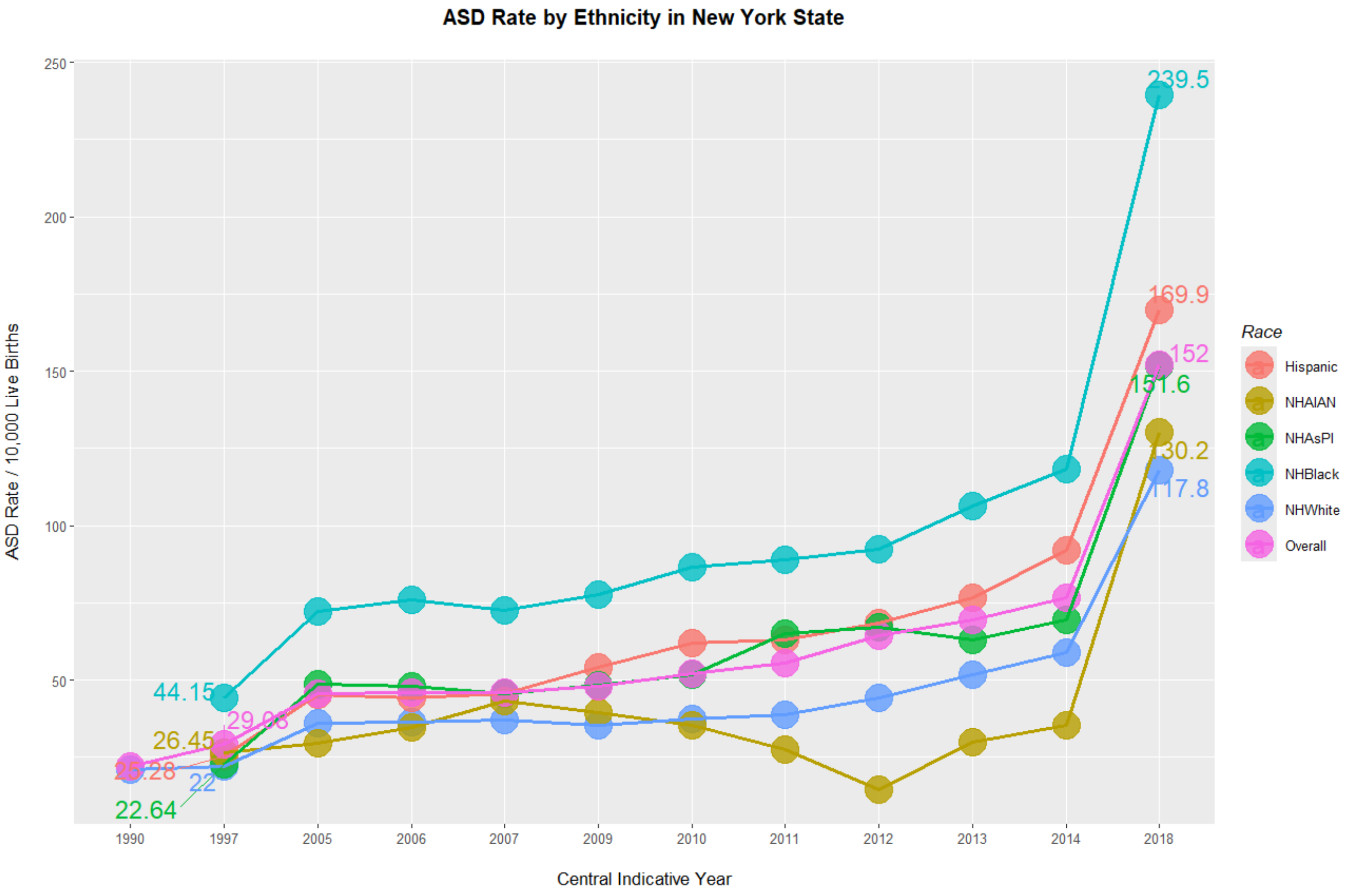

Figure S6.: ASD Rates in New York (More Details) by Ethnicity

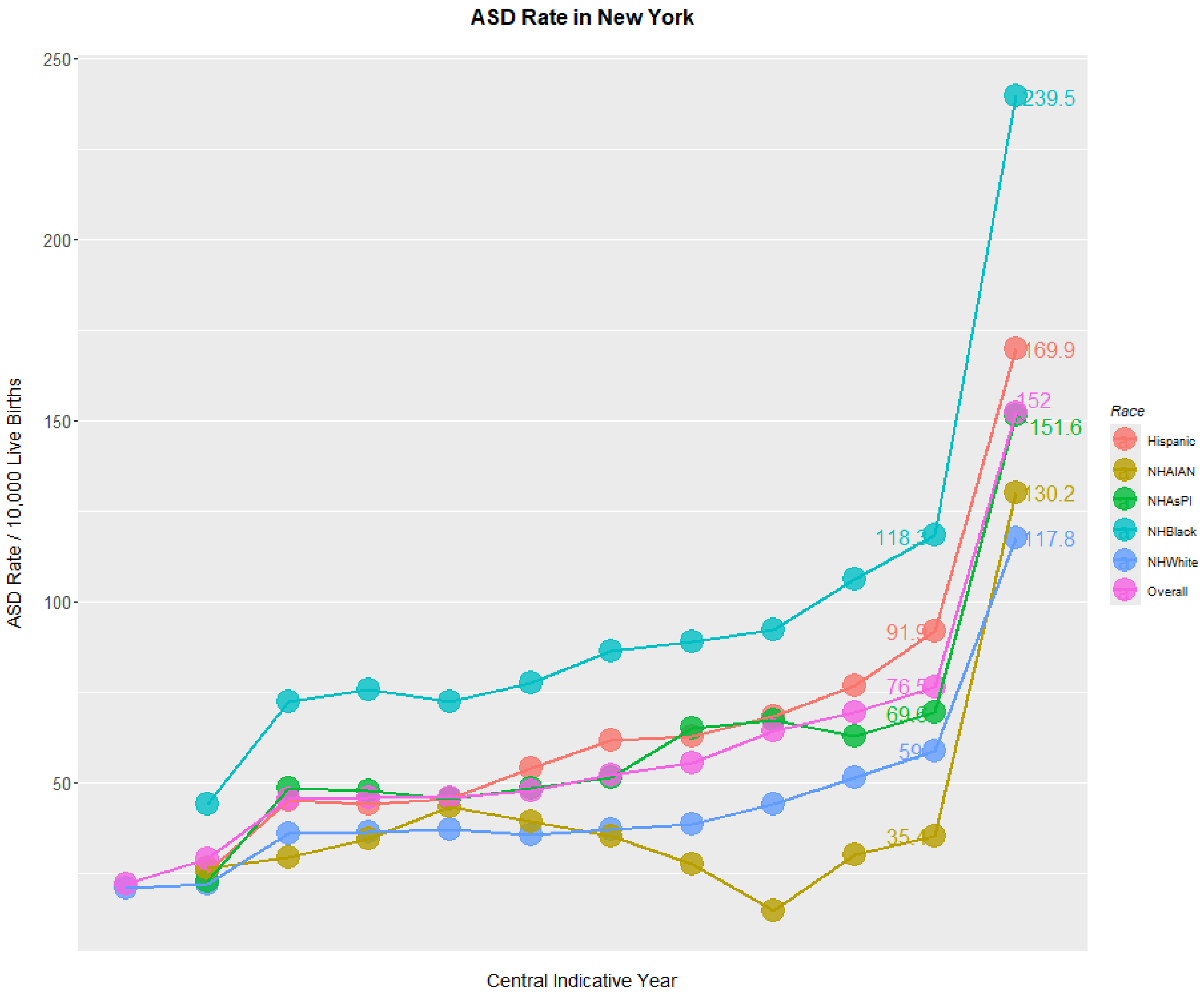

Figure S7.: ASD Rates in Department of Defence by Ethnicity

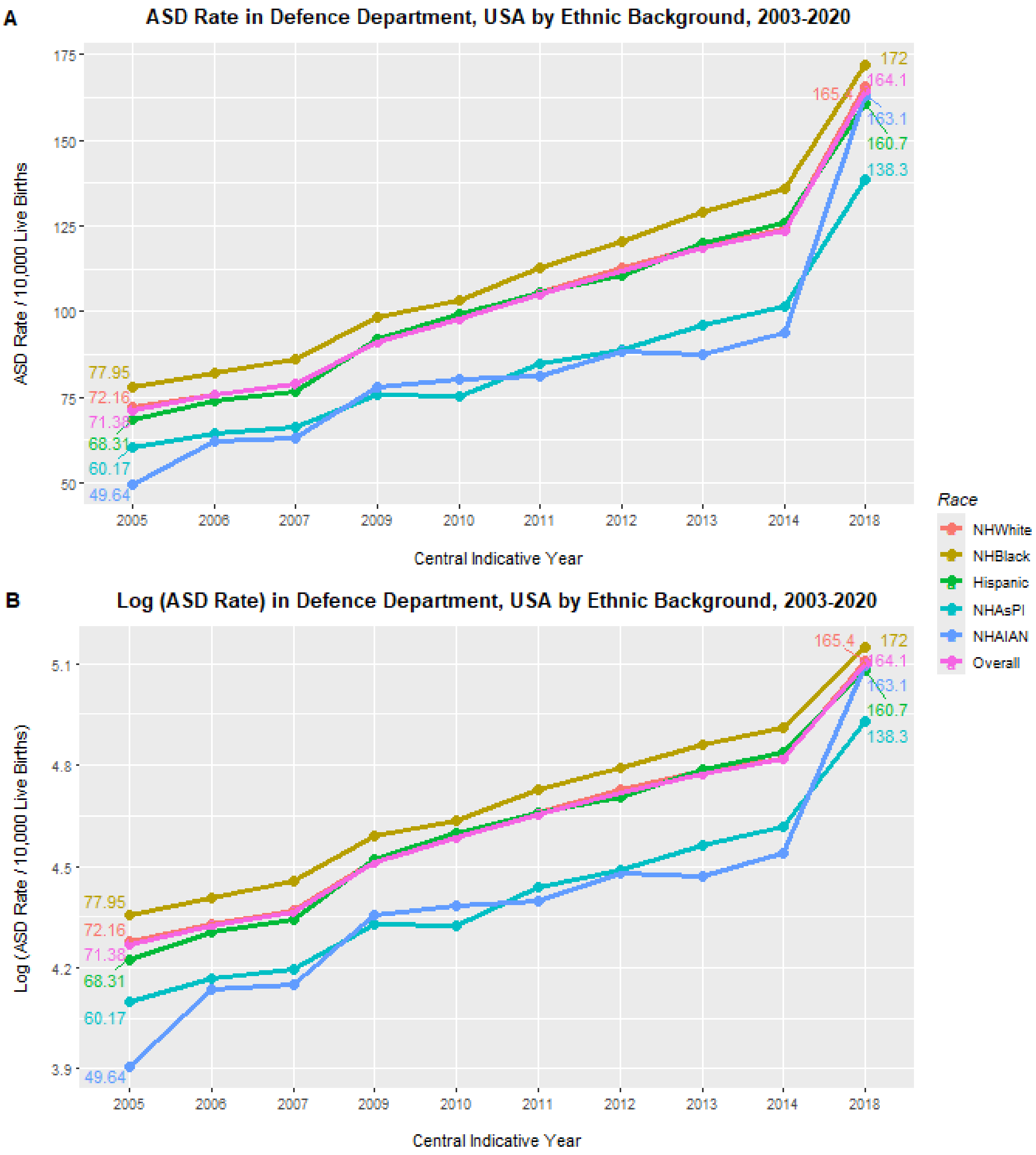

Figure S8.: ASD Rates in Kentucky by Ethnicity

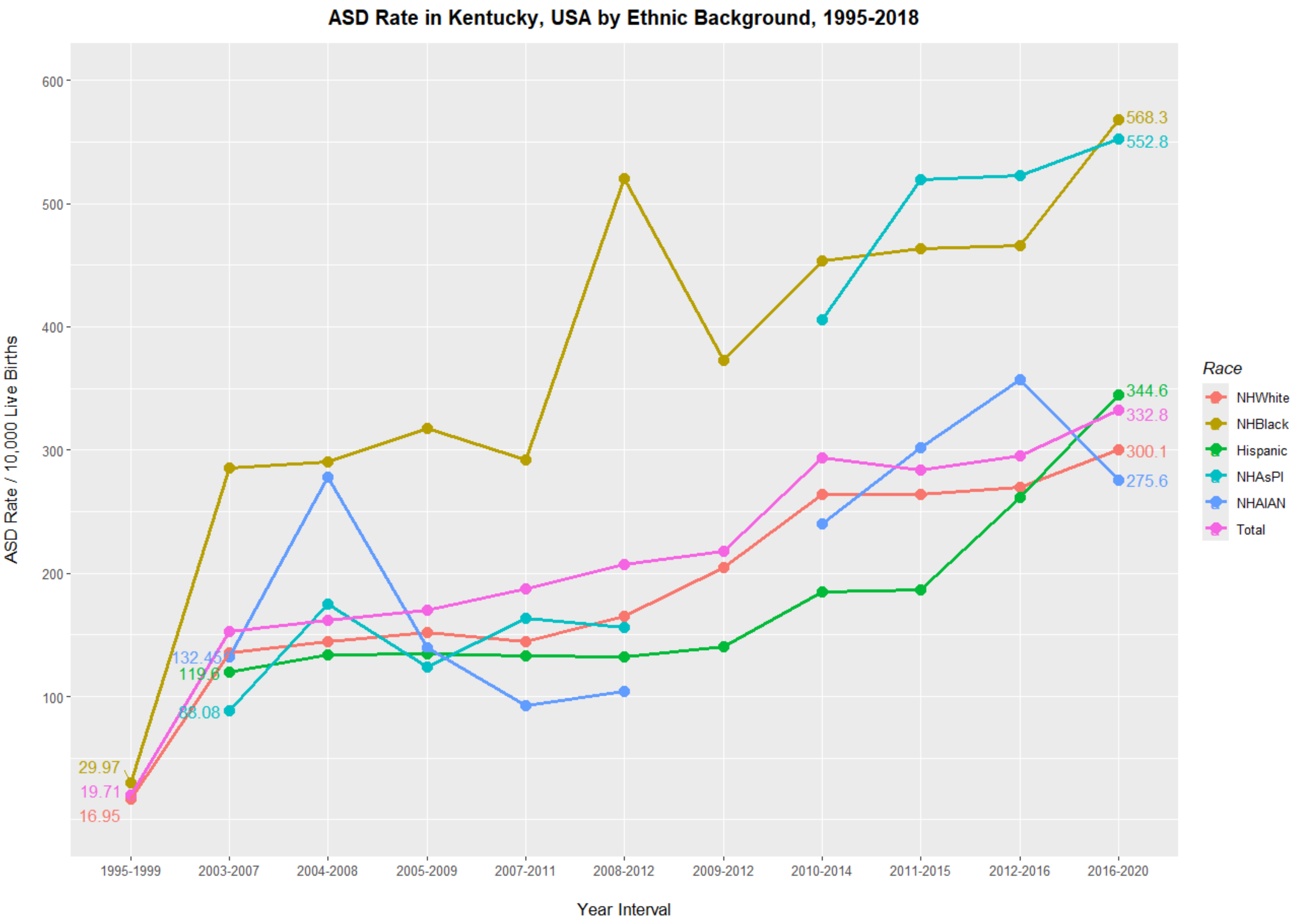

Figure S9.: ASD Rates in Michigan by Ethnicity

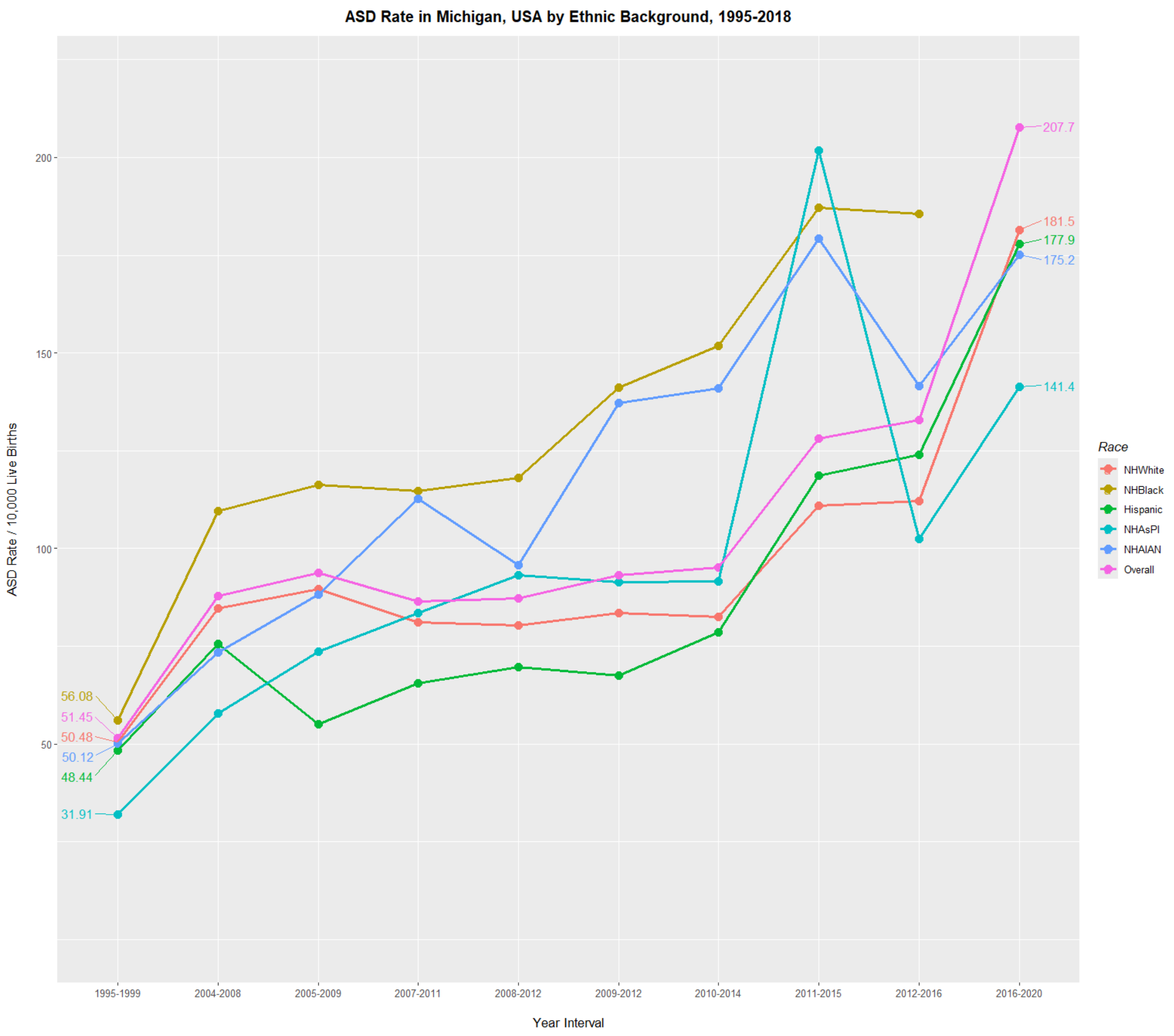

Figure S10.: ASD Rates in Colorado by Ethnicity

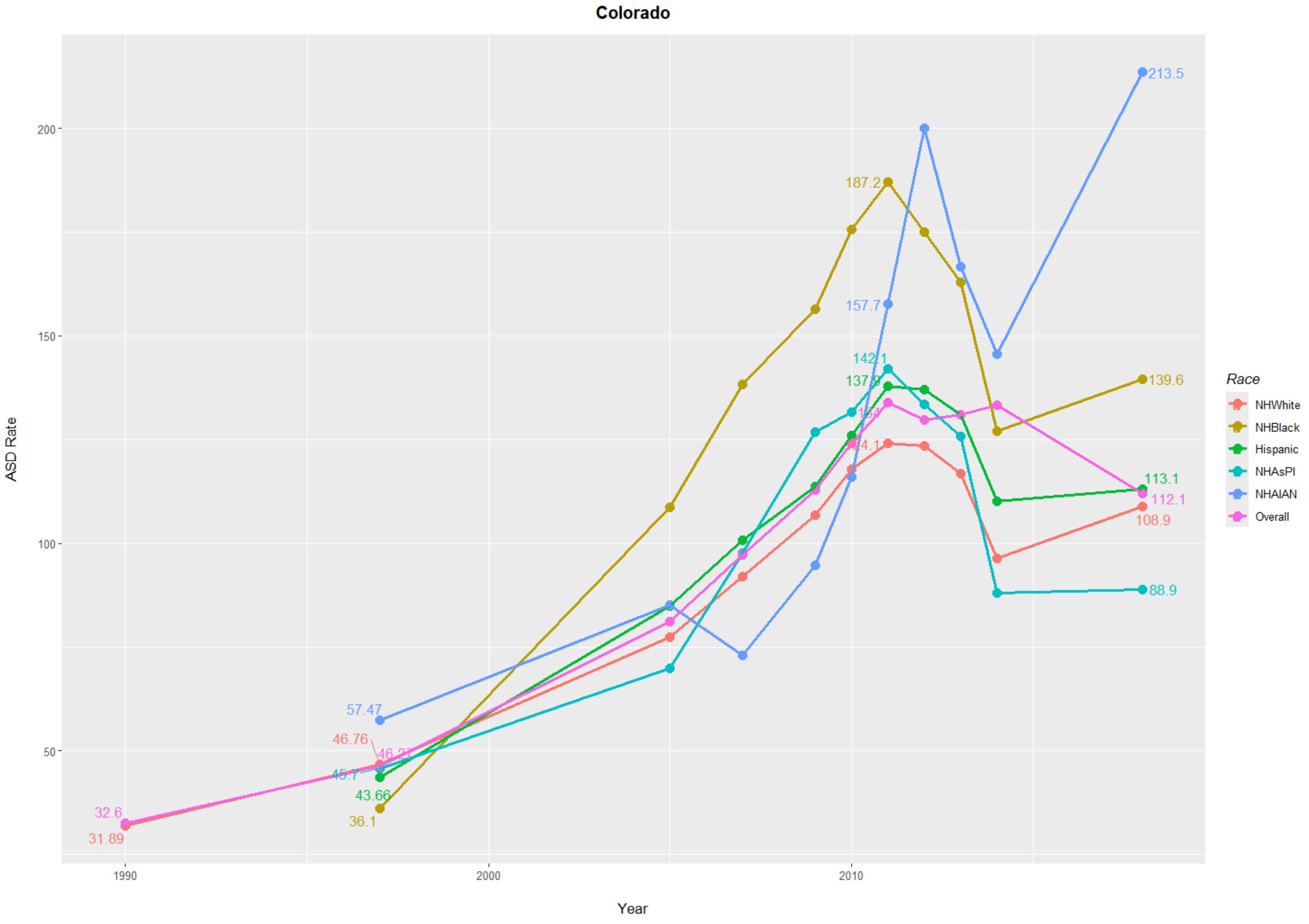

Figure S11.: ASD Rates in Tennessee by Ethnicity

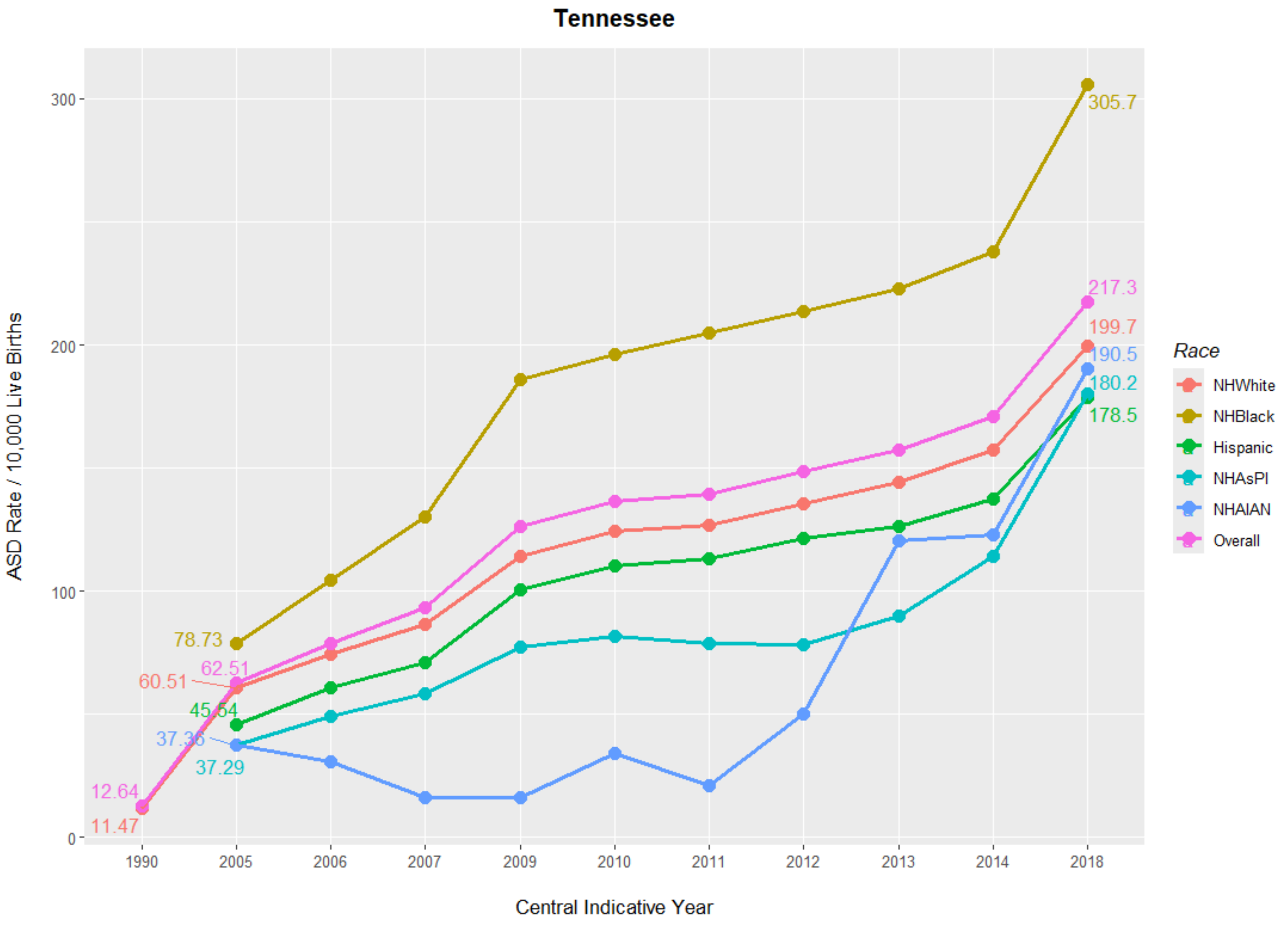

Figure S12.: ASD Rates in Alaska by Ethnicity

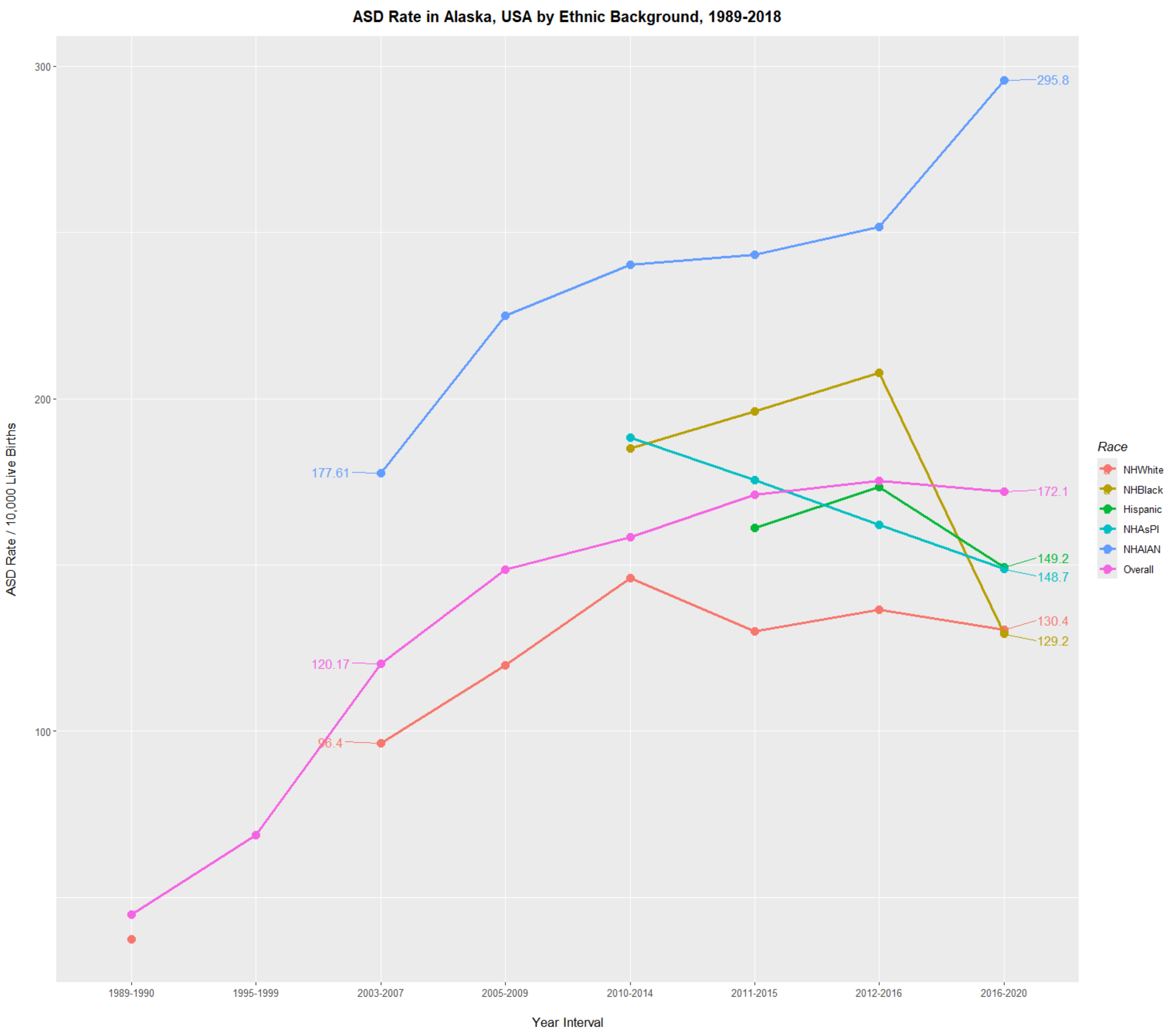

Figure S13.: ASD Rates in Mississippi by Ethnicity

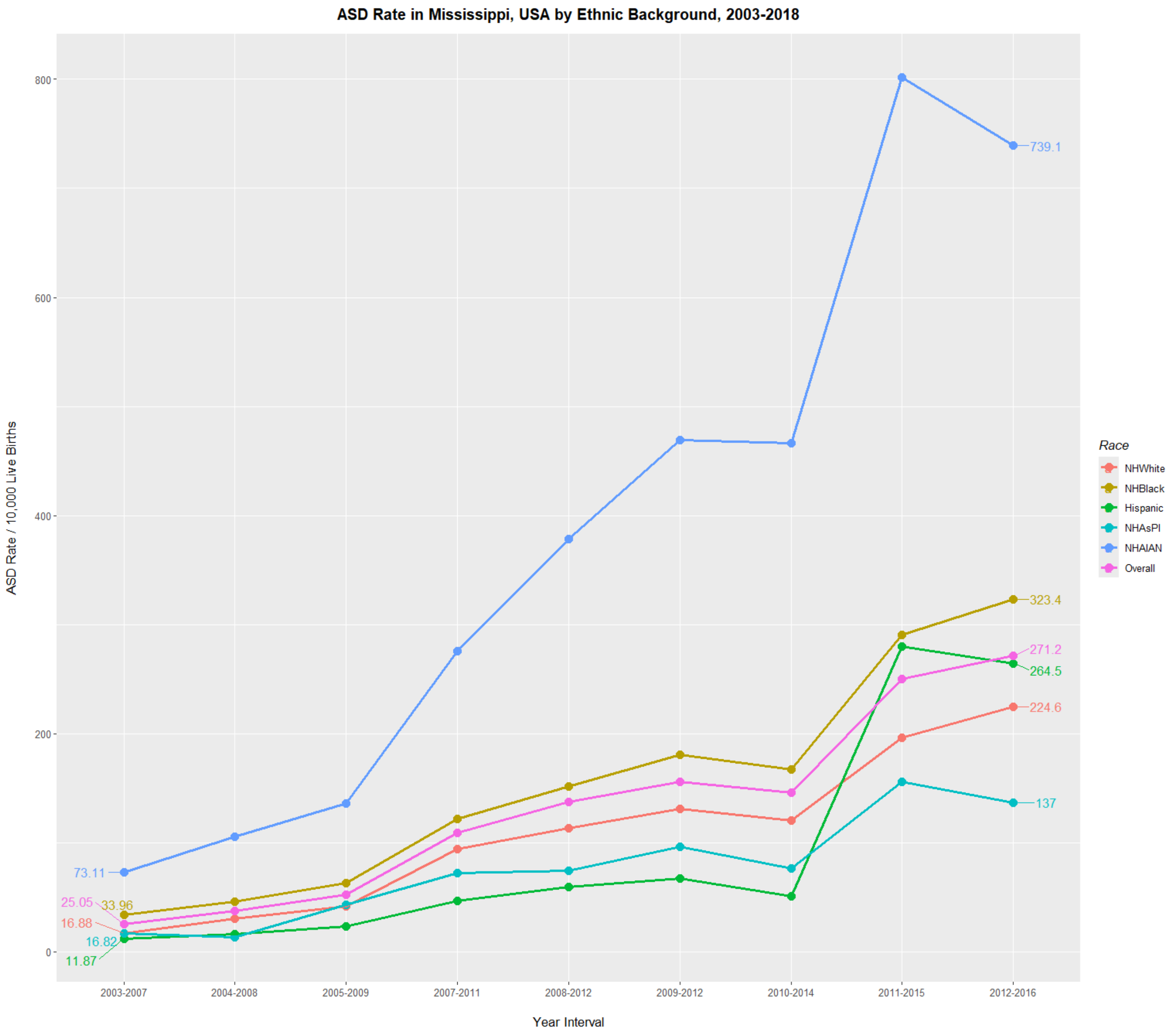

Figure S14.: ASD Rates in Missouri by Ethnicity

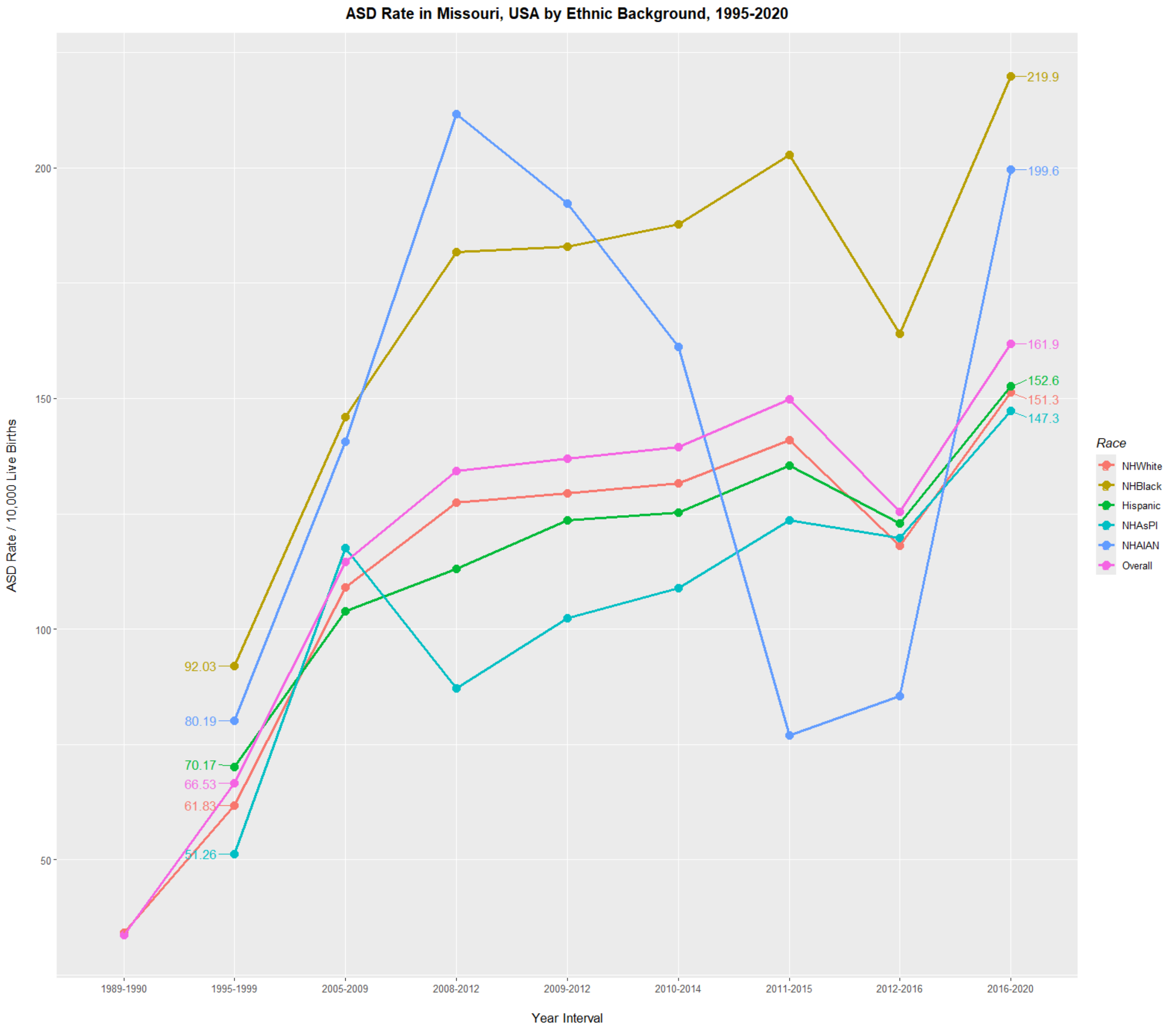

Figure S15.: ASD Rates in New Mexico by Ethnicity

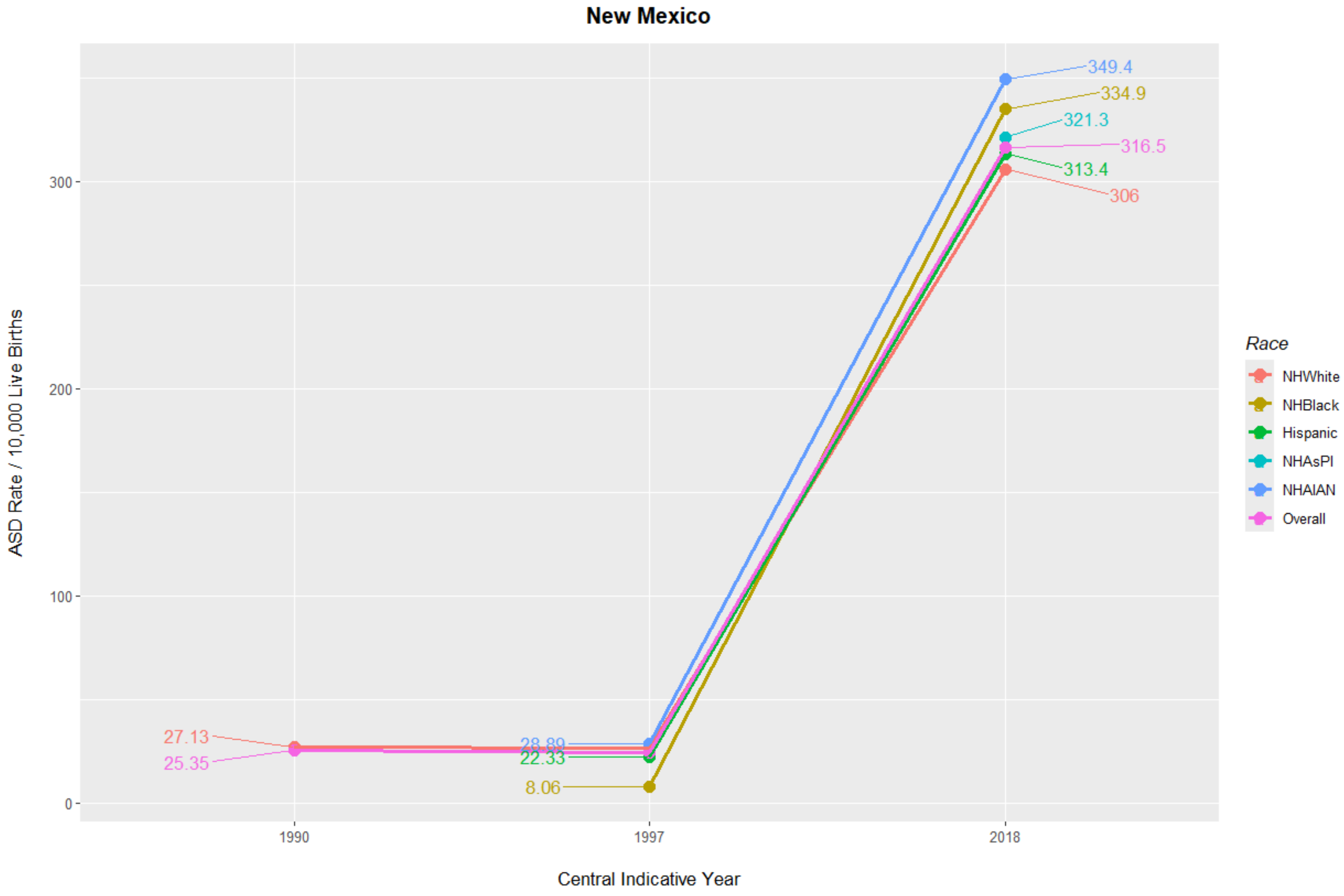

Figure S16.: ASD Rates in Oregon by Ethnicity

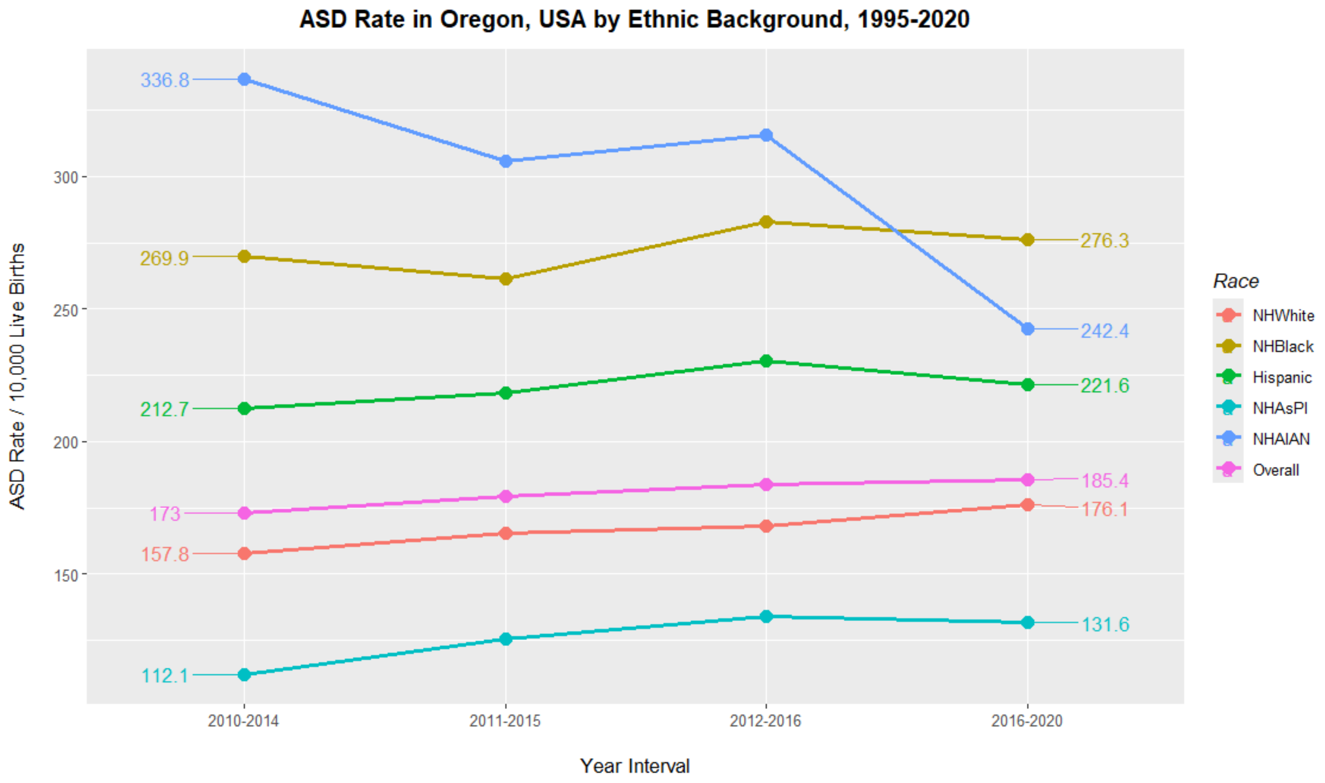

Figure S17.: ASD Rates in Florida by Ethnicity

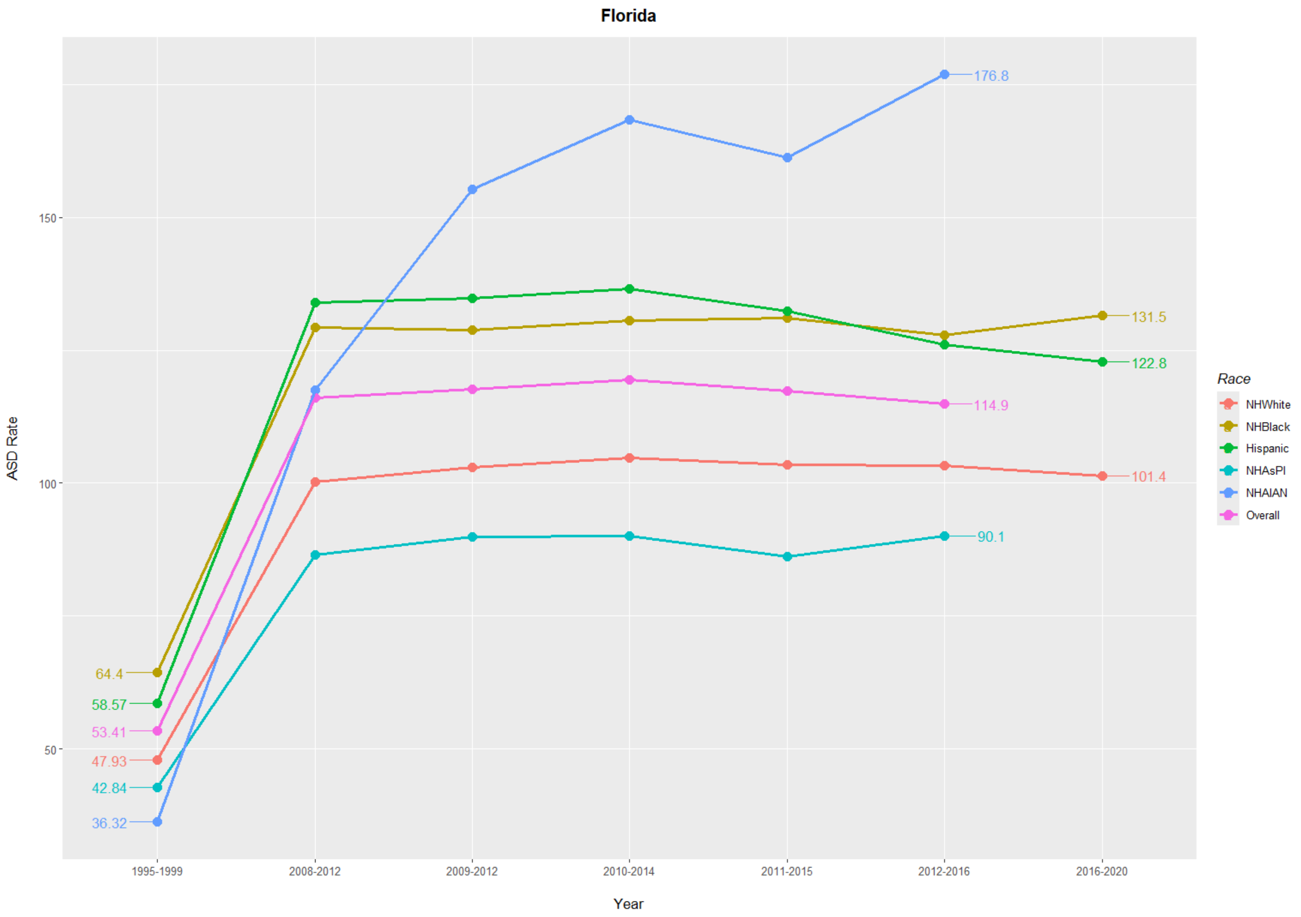

Figure S18.: ASD Rates in Utah by Ethnicity

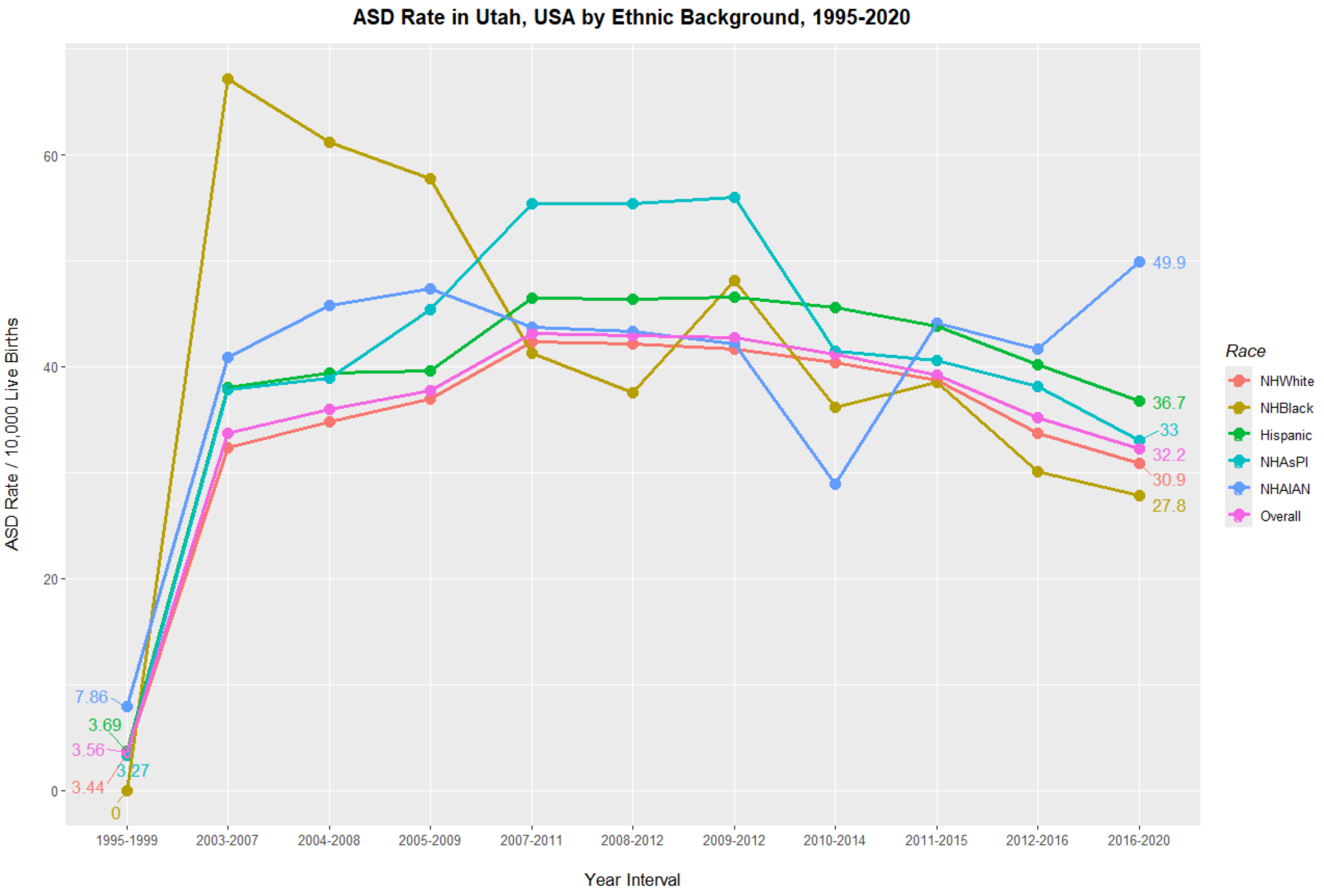

Figure S19.: ASD Rates in Texas by Ethnicity

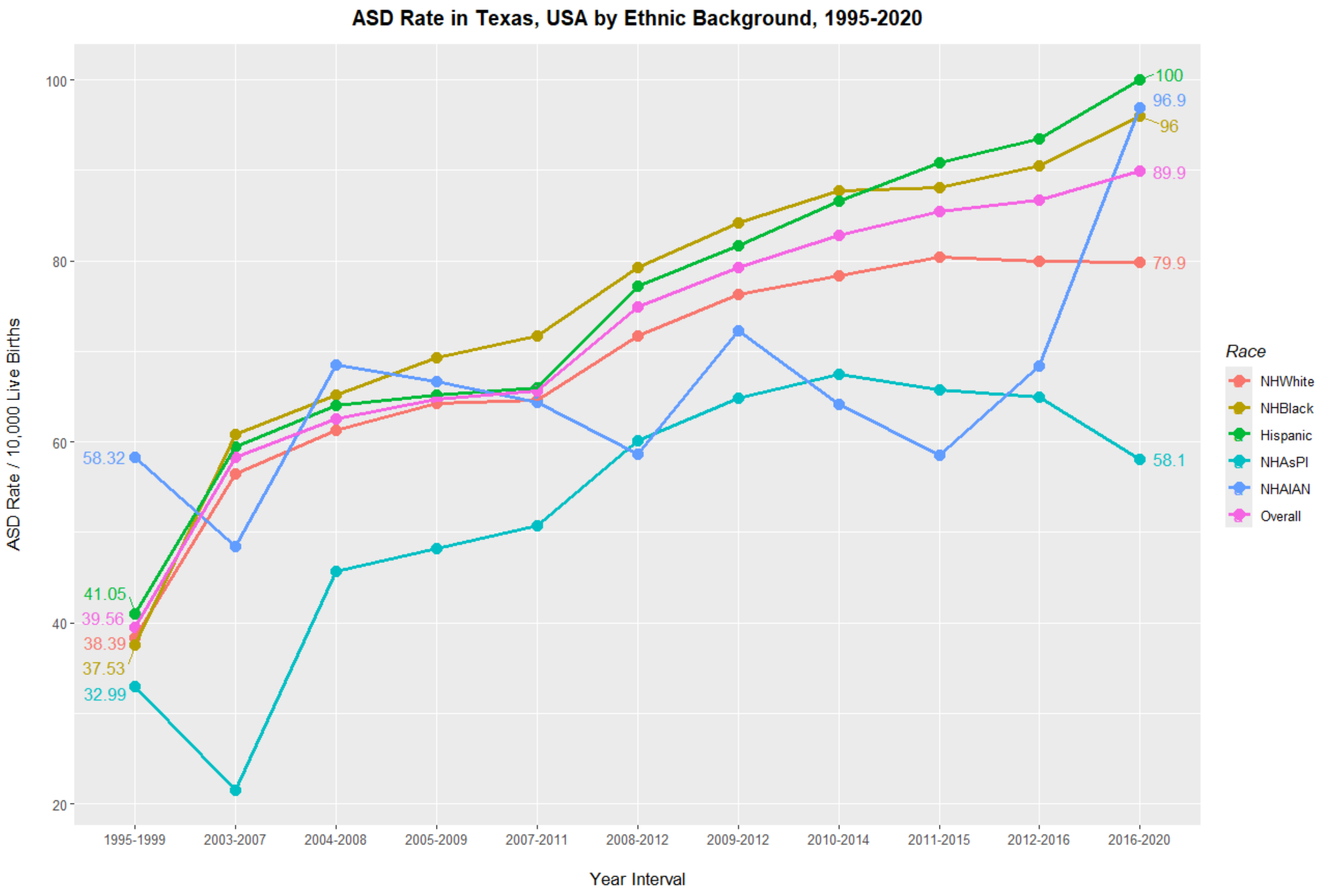

Figure S20.: ASD Rates in Georgia by Ethnicity

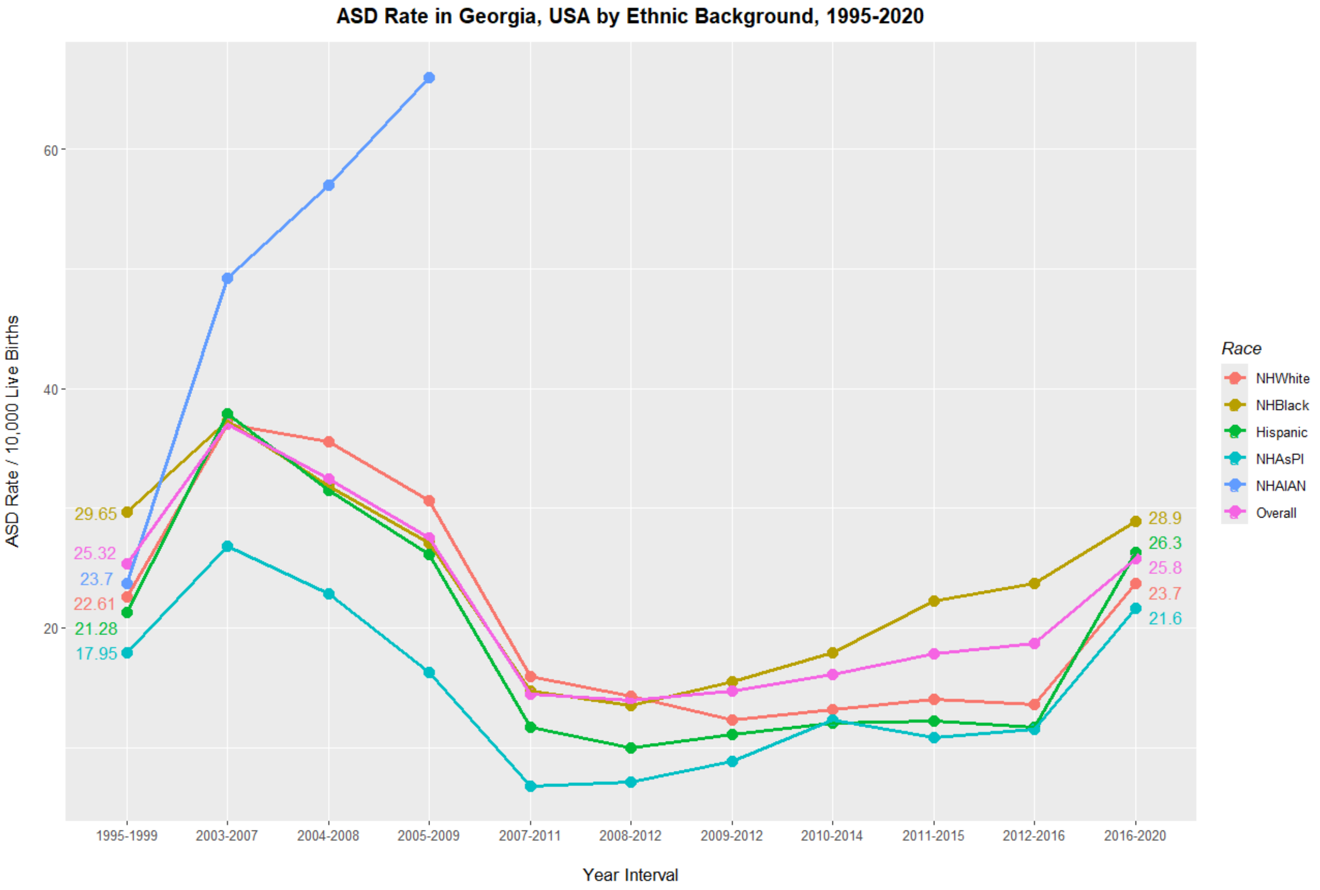

Figure S21.: ASD Rates in Maryland by Ethnicity

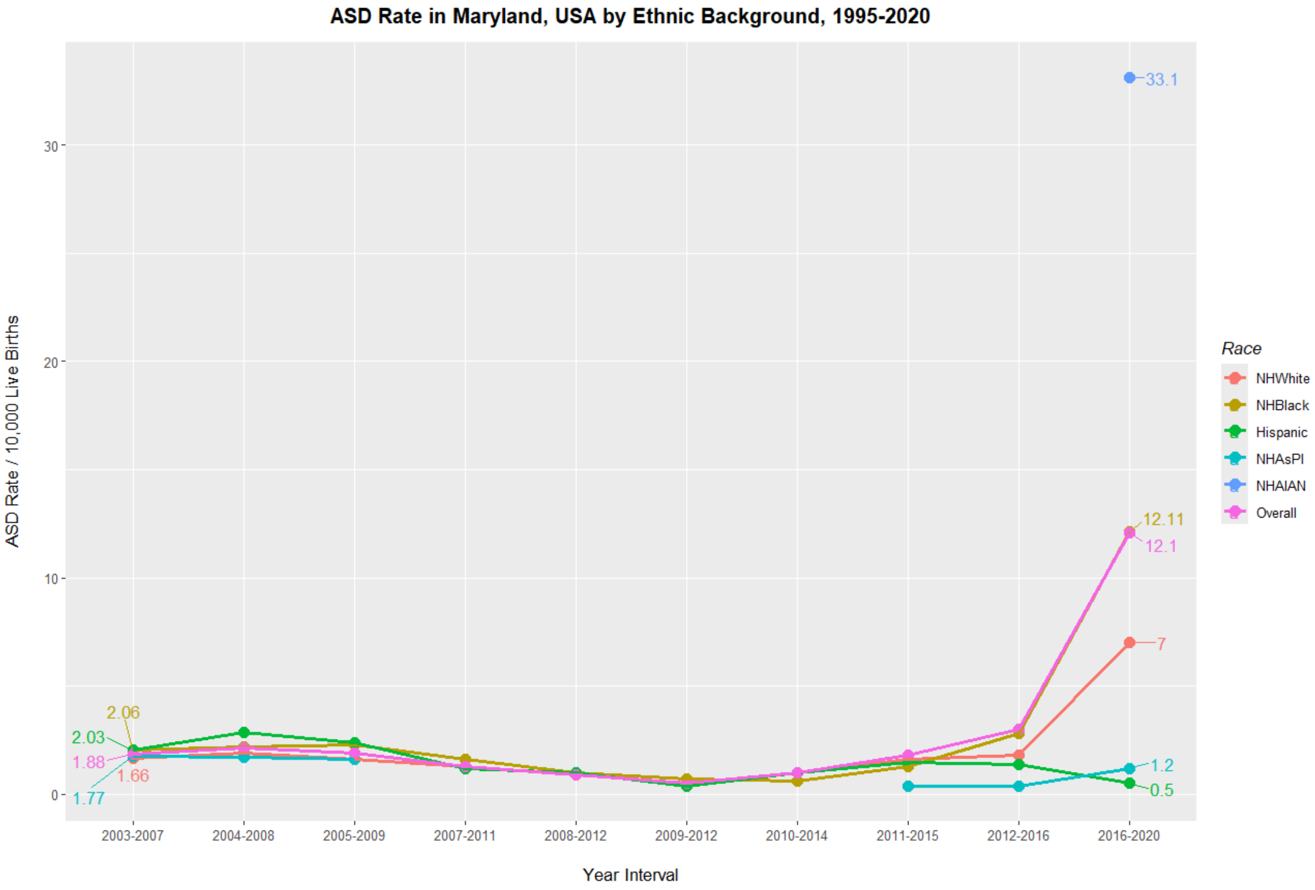

Figure S22.: ASD Rates in Minnesota by Ethnicity

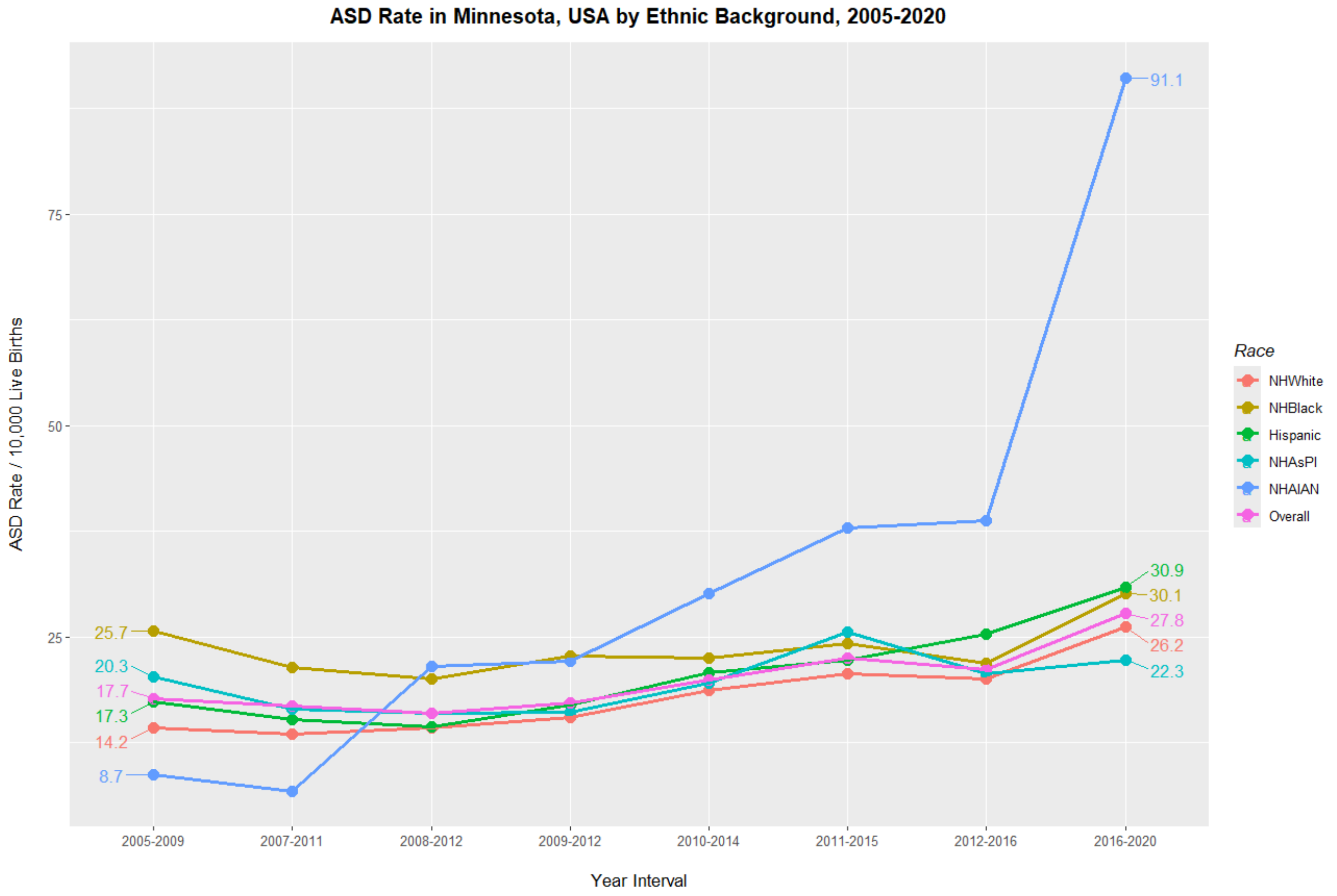

Figure S23.: ASD Rates in Iowa by Ethnicity

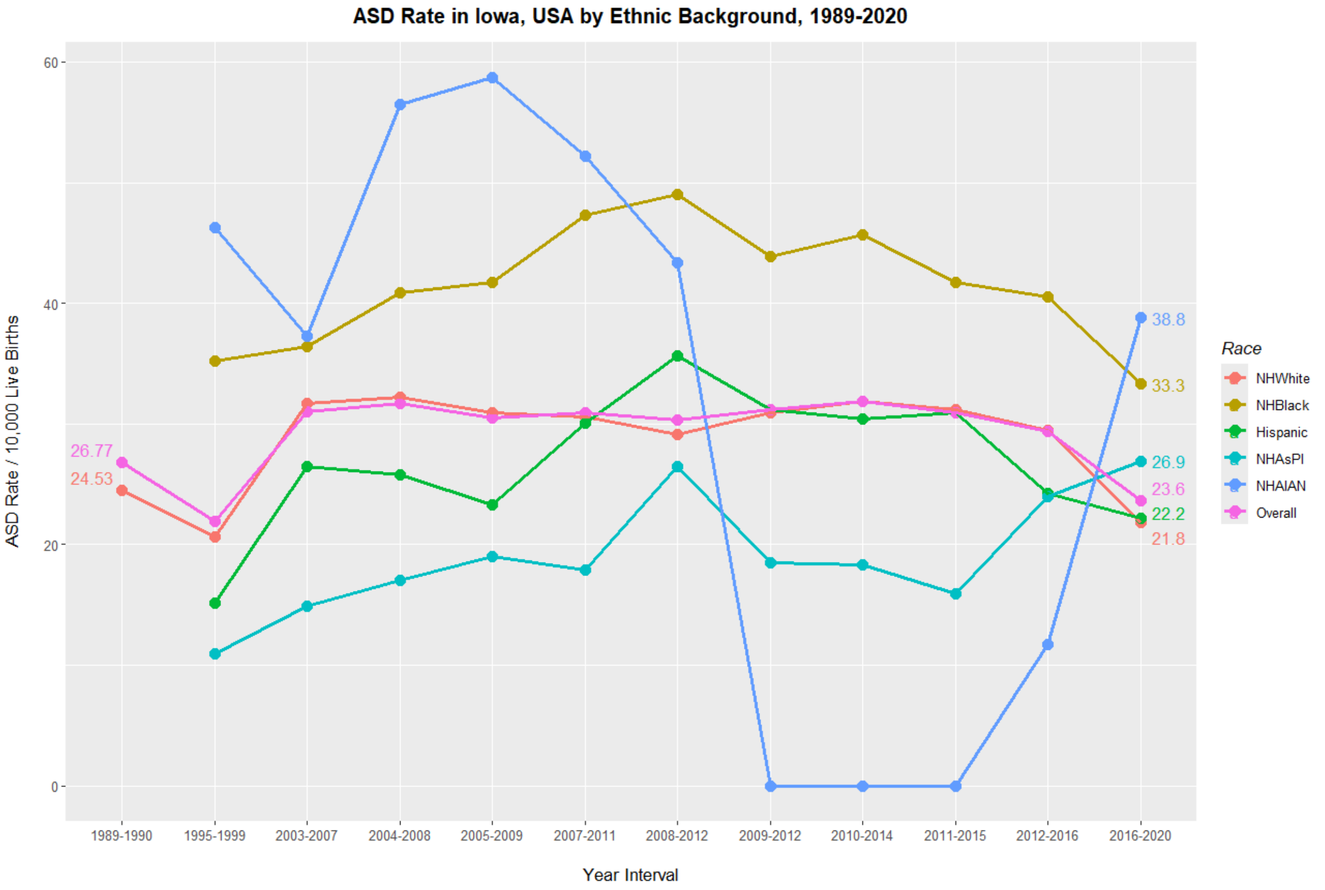

Figure S24.: ASD Rates in South Carolina by Ethnicity

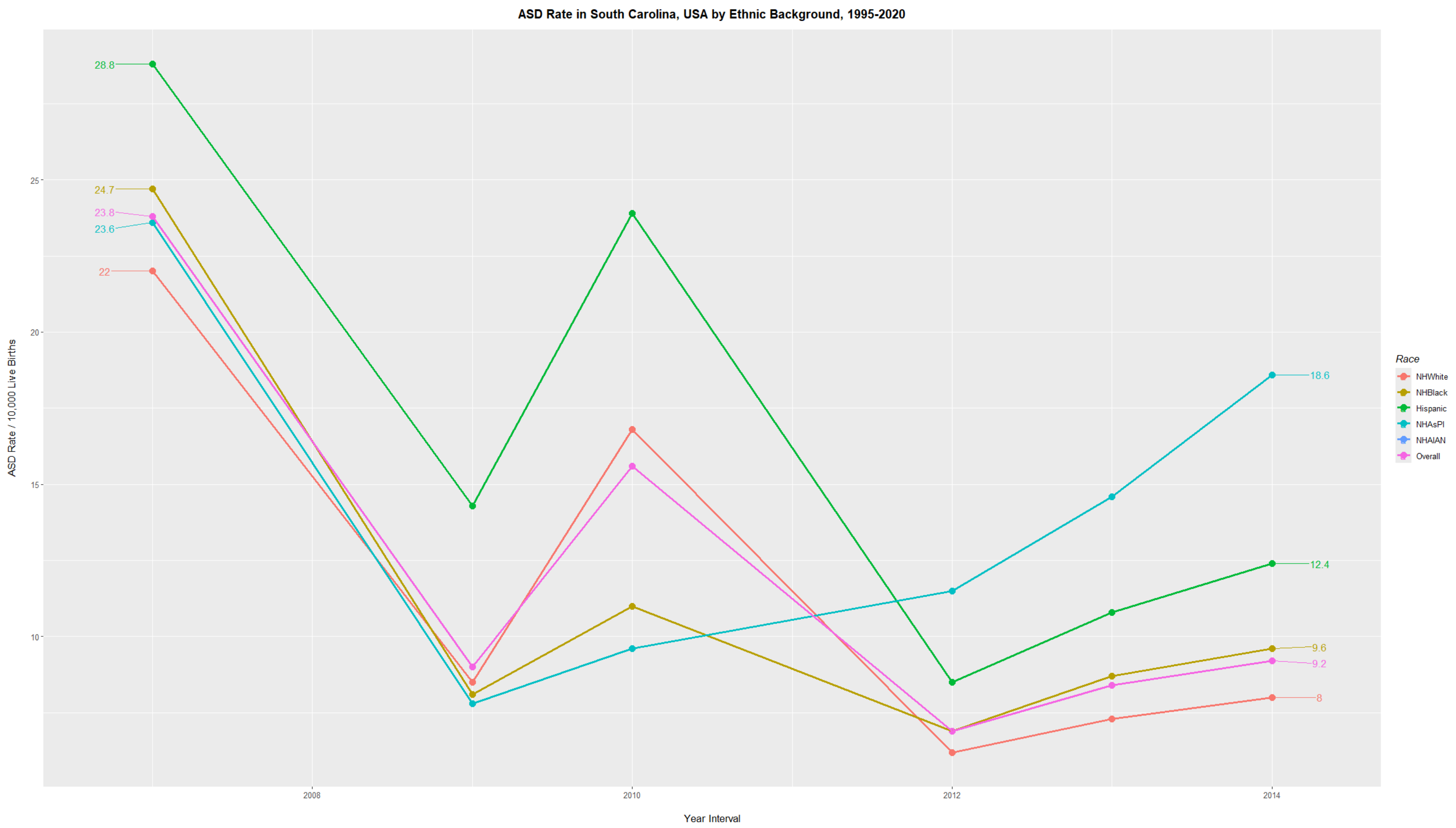

Figure S25.: ASD Rates in New Jersey by Ethnicity

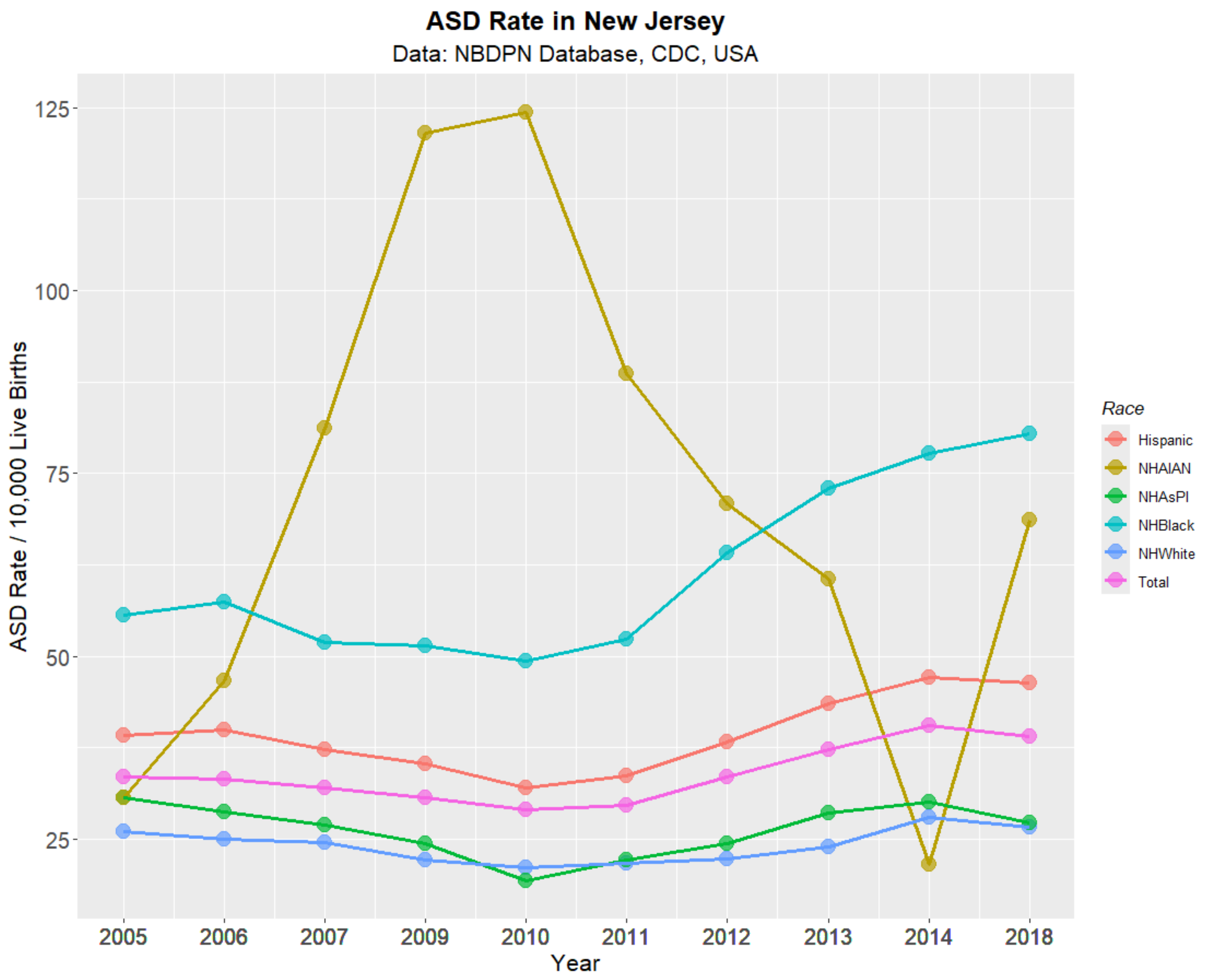

Figure S26.: Rates of Substance Exposure in USA

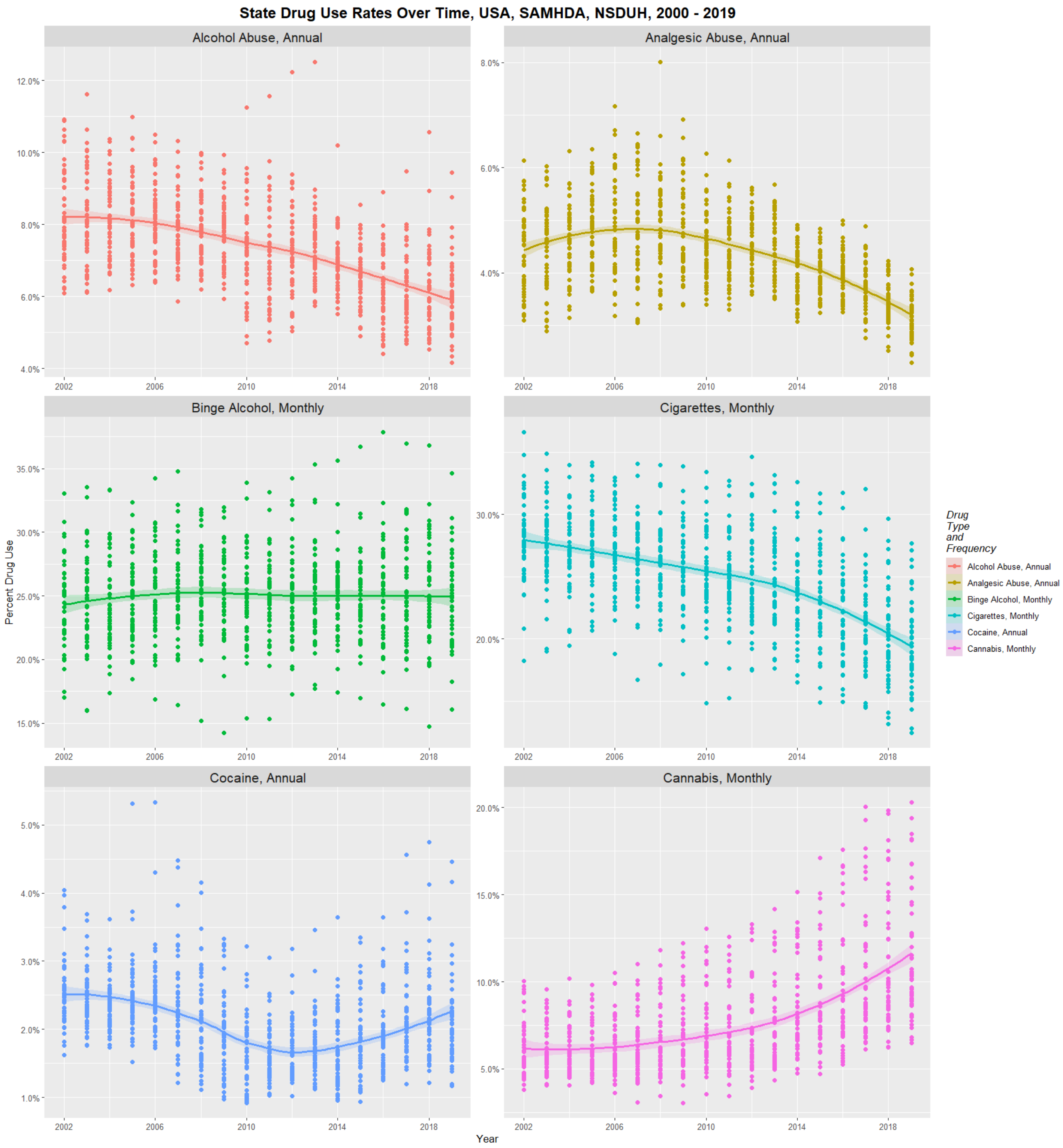

Figure S27.: ASD Rates by Selected Substances, Loess Lines

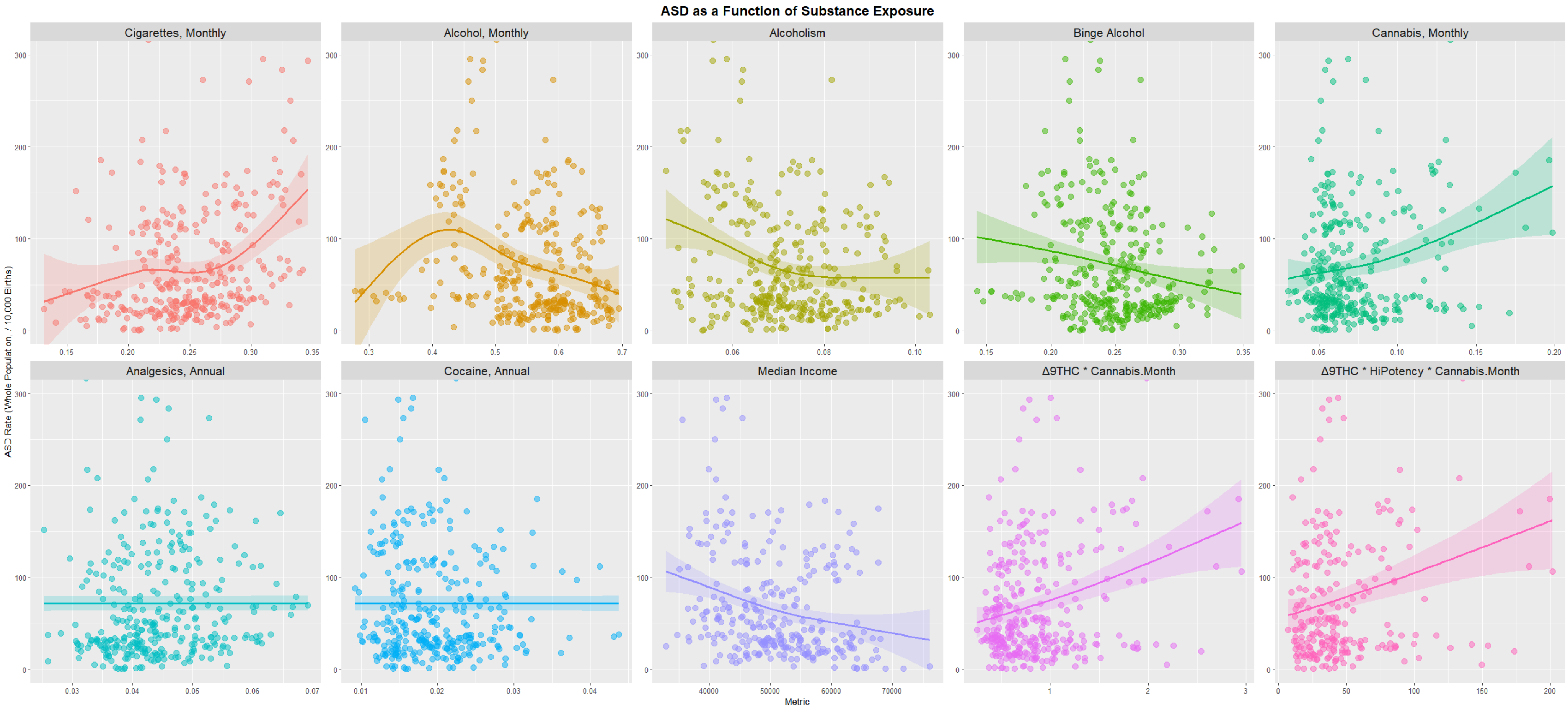

Figure S28.: ASD Rates by Selected Substances, Regression Lines

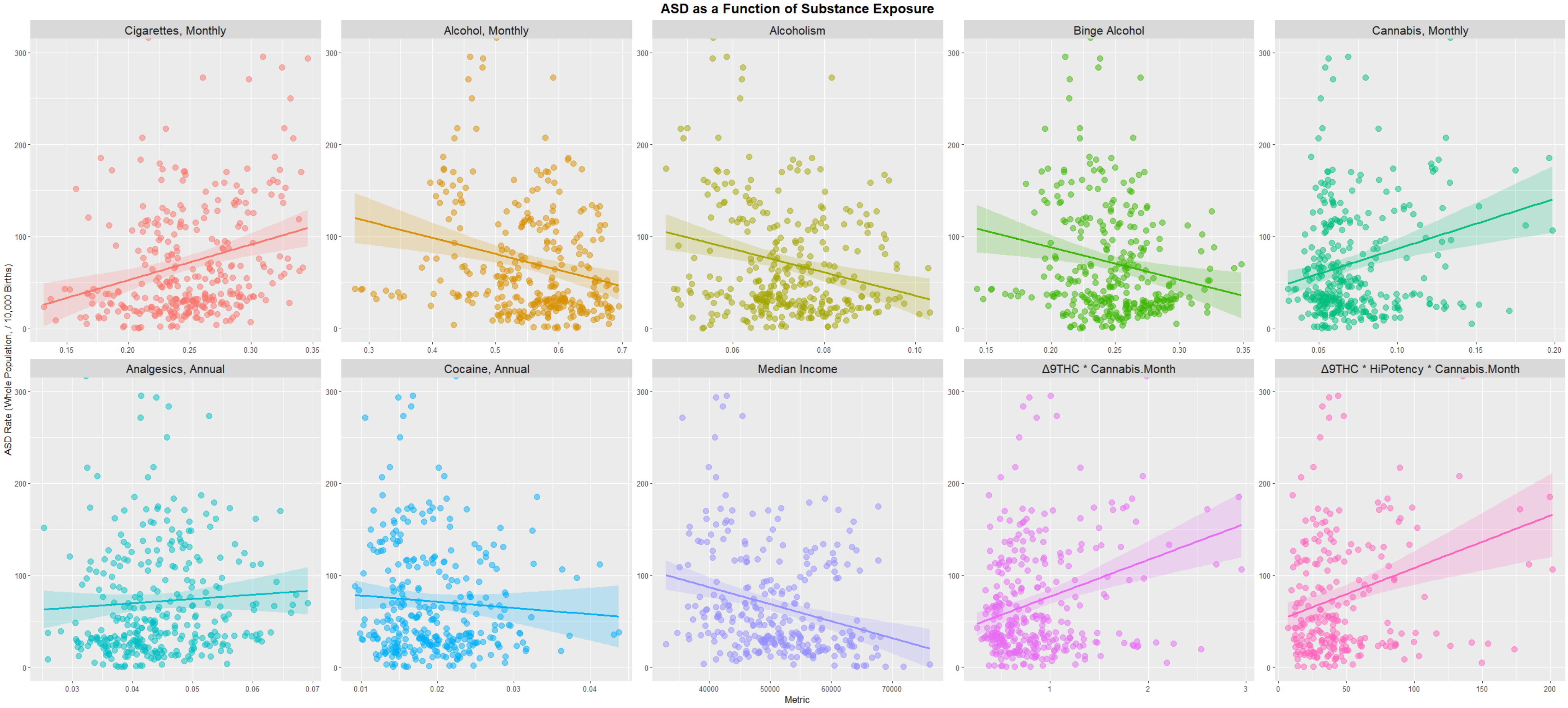

Figure S29.: Log (ASD Rates) by Selected Substances, Regression Lines

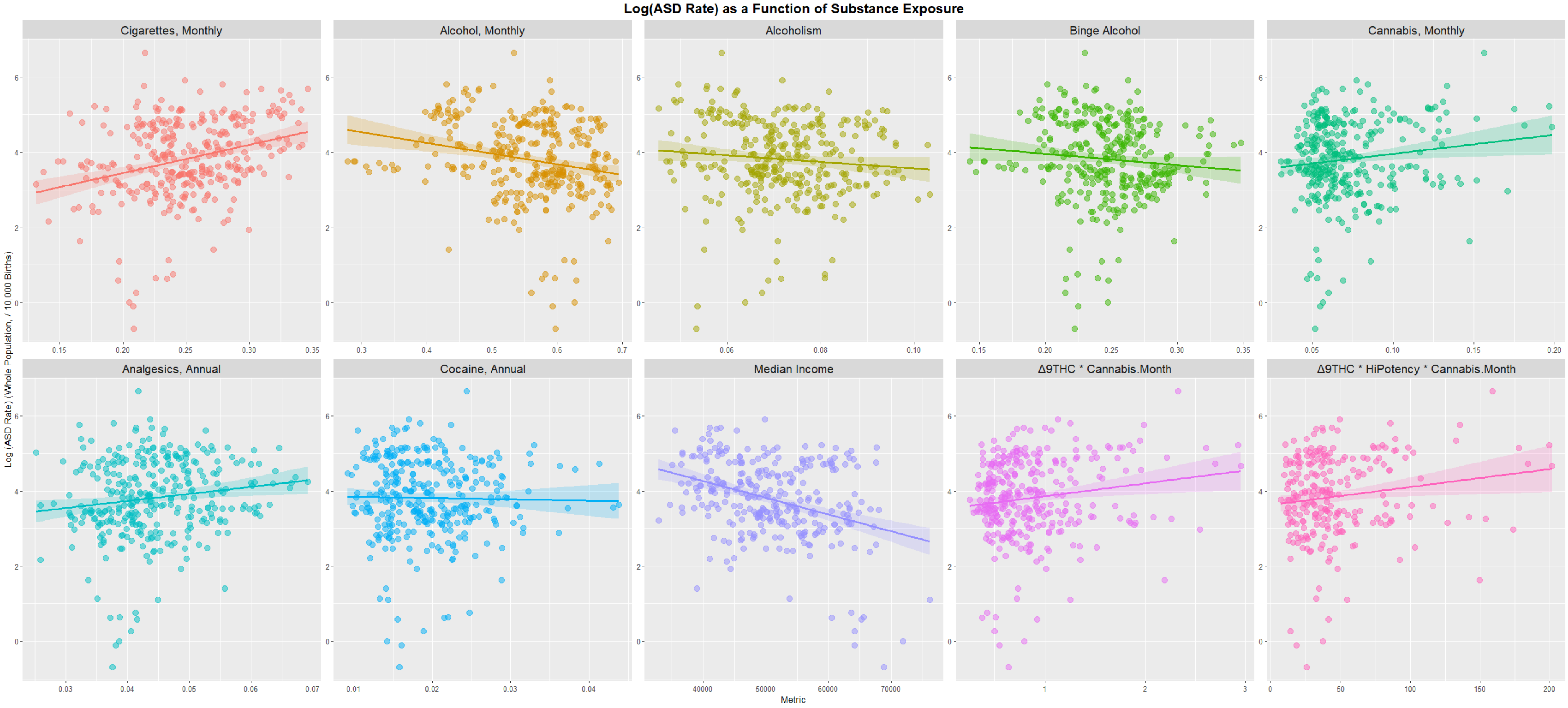

Figure S30.: Cannabinoid Exposure Trends Across USA

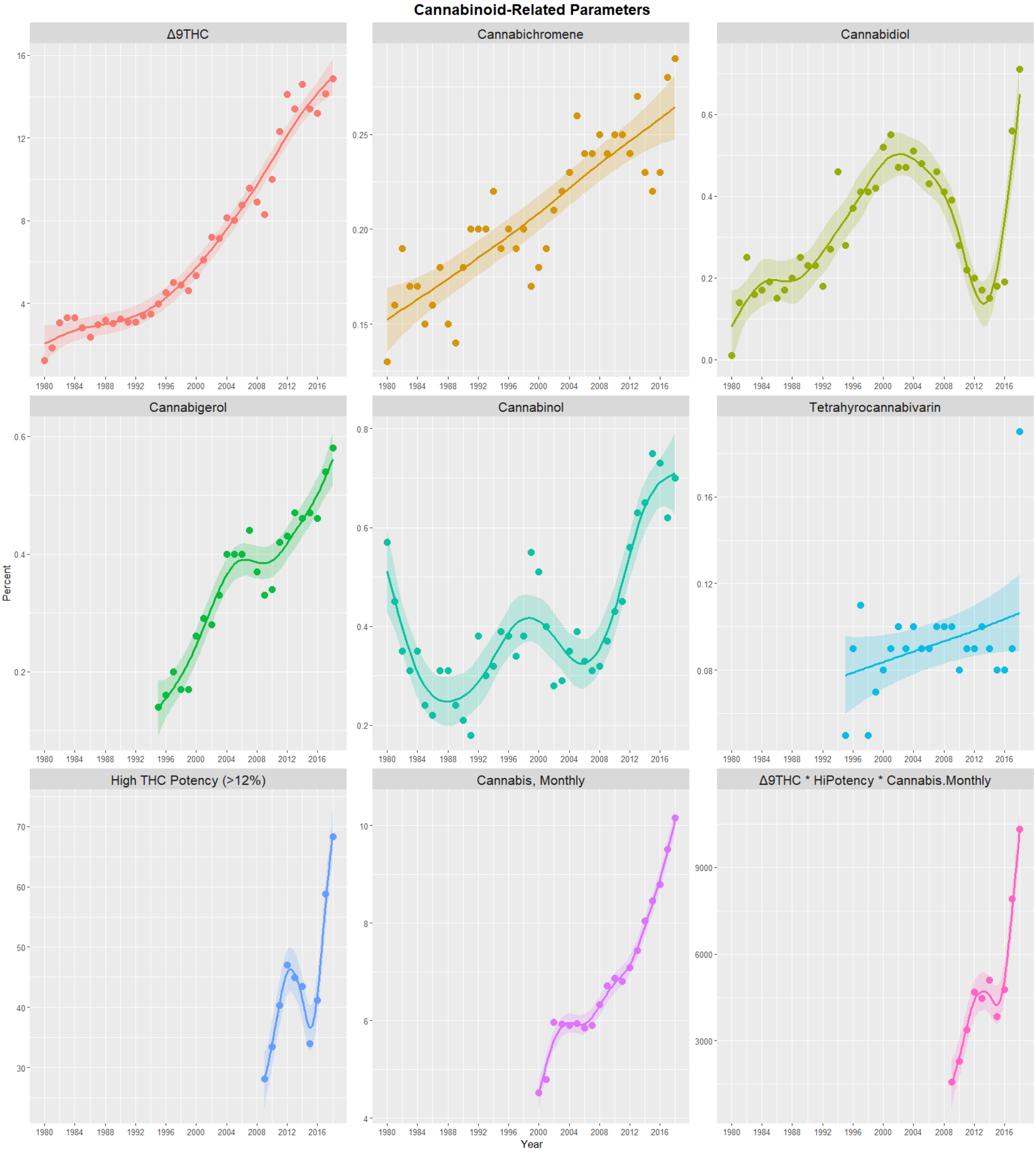

Figure S31.: Slopes ASD by Time v ASD by Cannabis Regression lines

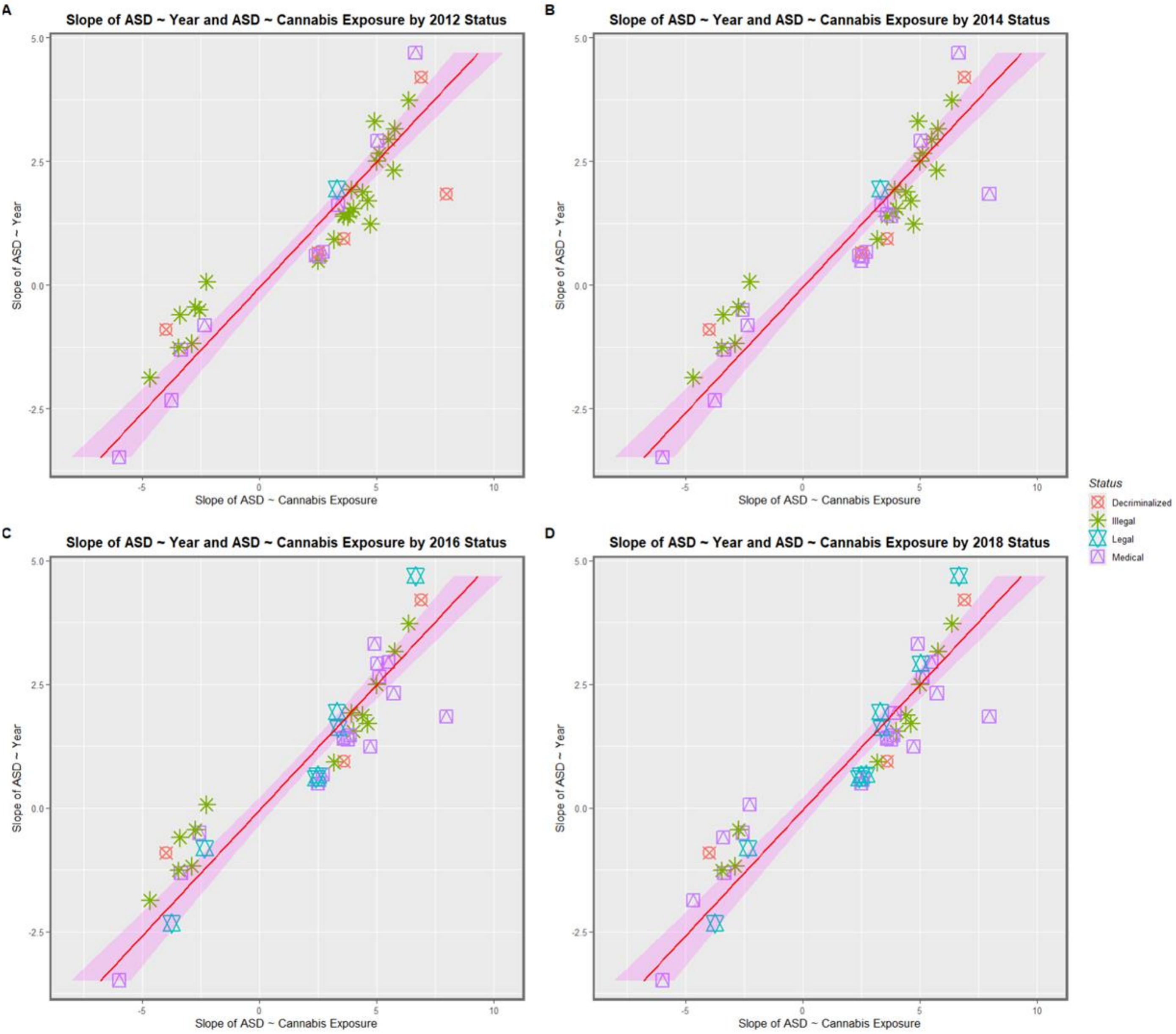

Figure S32.: Variable Importance Plot

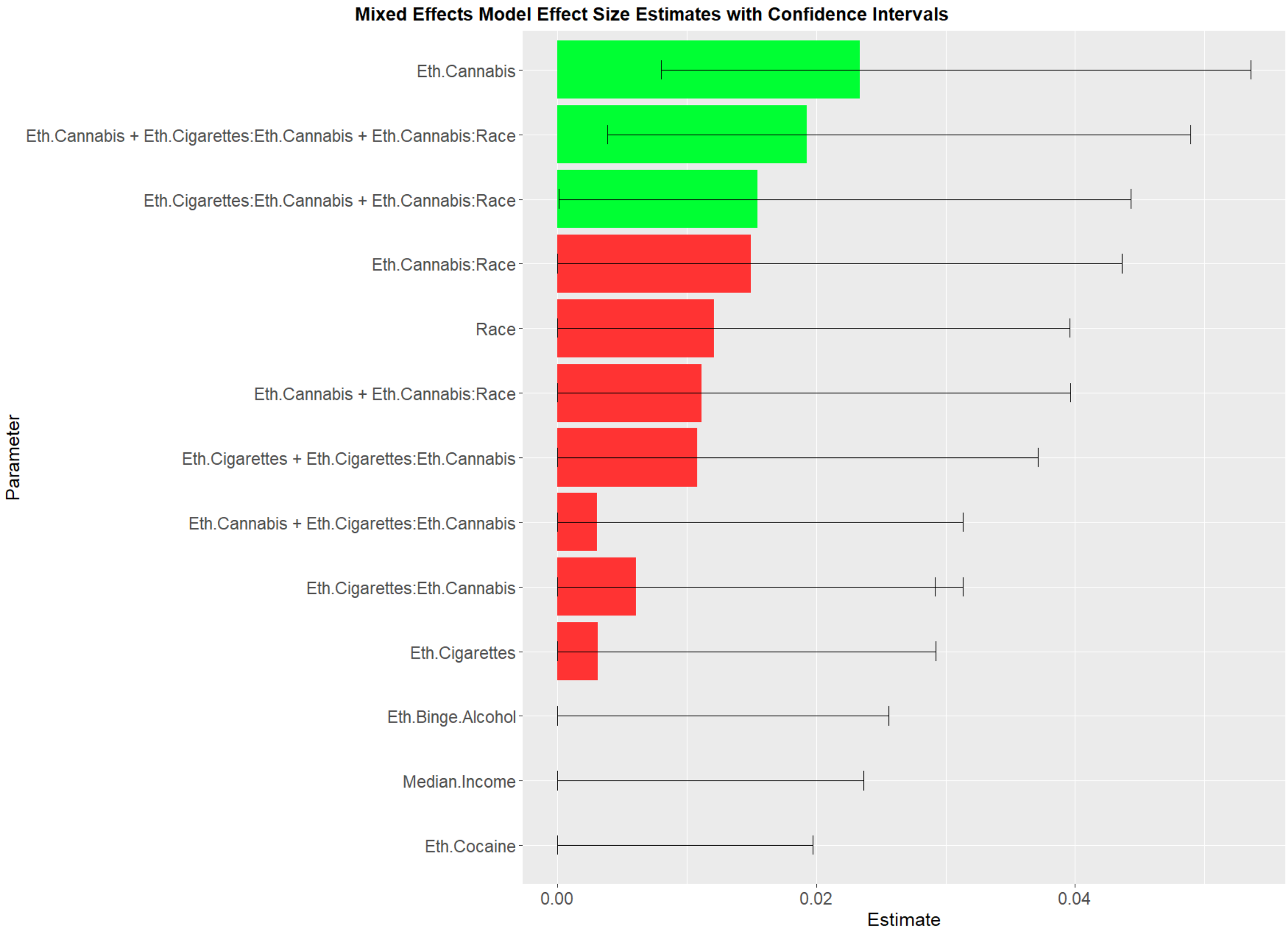

Figure S33.: Trends in Cannabis Legal Status Across USA States

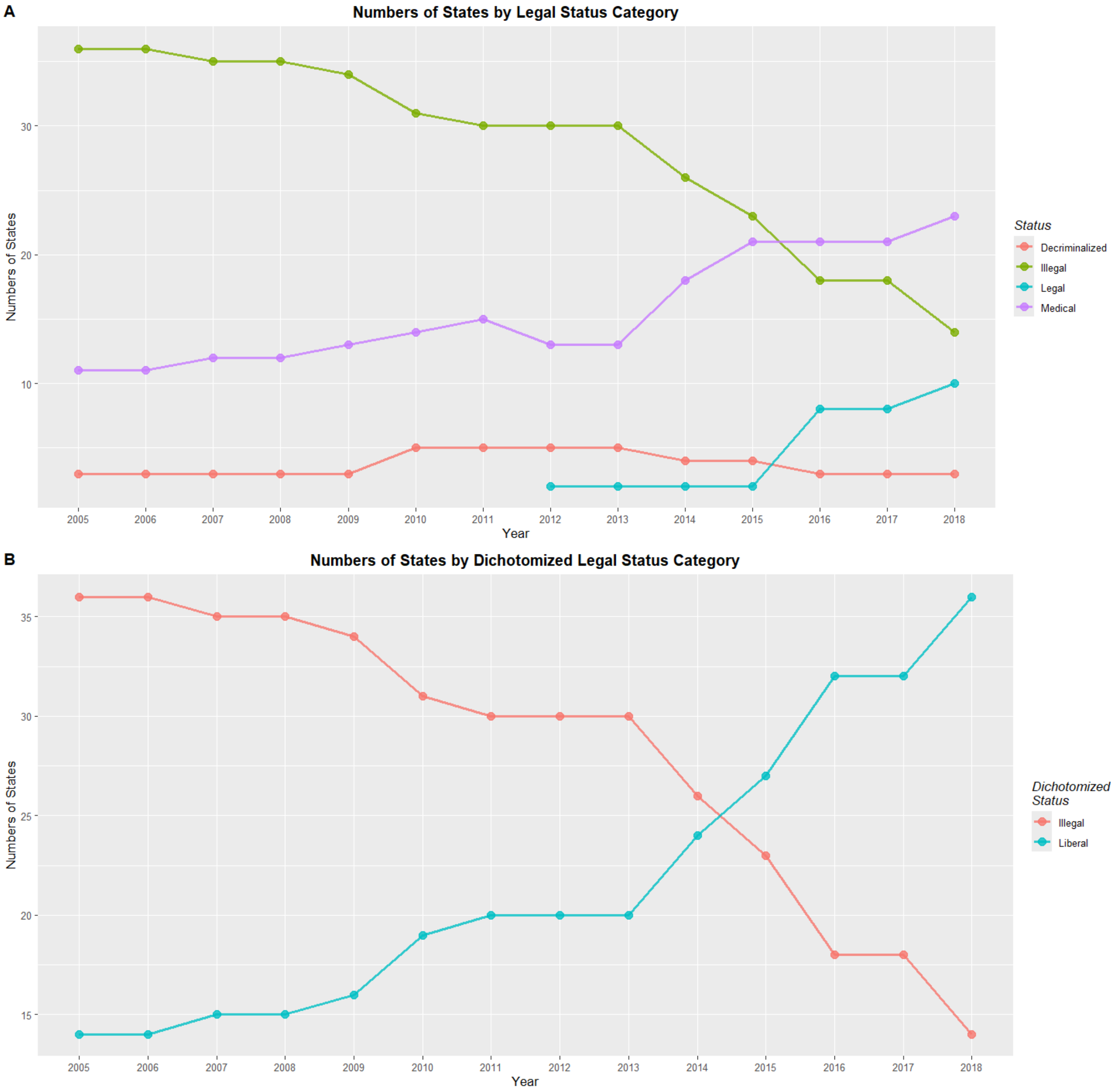

Supplement: Supplementary file 1 [file jox-16-00043-s001.zip › jox-3977265-supplementary.pdf]
